# Perylene dianhydride hydrogels obtained from accessible perylene diamic acid salts by a versatile protonation–hydrolysis mechanism†

**DOI:** 10.1039/d5ra00372e

**Published:** 2025-04-02

**Authors:** Markus C. Kwakernaak, Marijn Koel, Peter J. L. van den Berg, Nicolaas Strik, Wolter F. Jager

**Affiliations:** a Department of Chemical Engineering, Delft University of Technology Van der Maasweg 9 2629 HZ Delft The Netherlands w.f.jager@tudelft.nl; b Department of Radiation Science and Technology, Delft University of Technology Mekelweg 15 2629 JB Delft The Netherlands

## Abstract

Perylene dianhydride (PDA) hydrogels are made from highly accessible perylene diamic acid salts (PDAA salts) by a versatile protonation–hydrolysis mechanism. Very weak gels are formed initially by π-stacking of PDAAs and subsequent hydrolysis yields much stronger PDA hydrogels. Hydrogels are readily made at concentrations down to 0.5 mM, exhibit storage moduli around 600 Pa @ 1 mM and undergo significant syneresis in time.

## Abstract 2

We report on the synthesis and gel forming abilities of perylene diamic acid salts, a new type of perylene hydrogelator that forms perylene dianhydride gels by a versatile protonation–hydrolysis mechanism.

## Graphical abstract

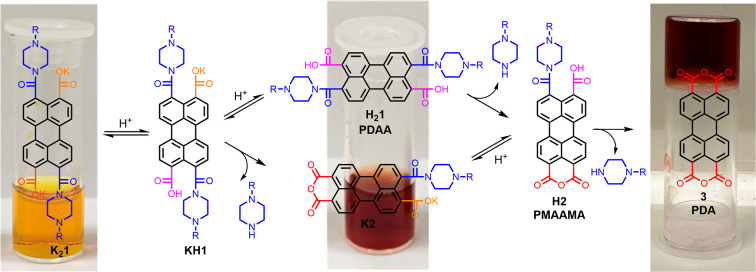

## Introduction

Perylene-3,4,9,10-teracarboxylic acid derivatives (PTCAs) form a class of aromatic dyes and pigments, of which perylene-3,4,9,10-tetracarboxylic diimides (PDIs) are the most abundantly exploited representatives.^1^ PDIs are produced on an industrial scale and applied as dyes and pigments.^2^ Because of their benign optoelectronic properties, potential applications in photovoltaics,^3^ artificial photosynthesis,^4^ photocatalysis^5^ and organic batteries^6^ are envisaged. Due to their hydrophobic nature and tendency to self-assemble by π-stacking,^7^ PDIs have been employed as gelators, both in water^8^ and in apolar solvents, where H-bonding complements the self-assembly process.^9^ Well-known examples of hydrogelators are amino acid-appended PDIs,^10^ that form gels upon protonation of their salts. In some cases the chirality of the appended amino acids is expressed in the ordering of the PDI molecules in the gel fibres.^11^

**Scheme 1:**
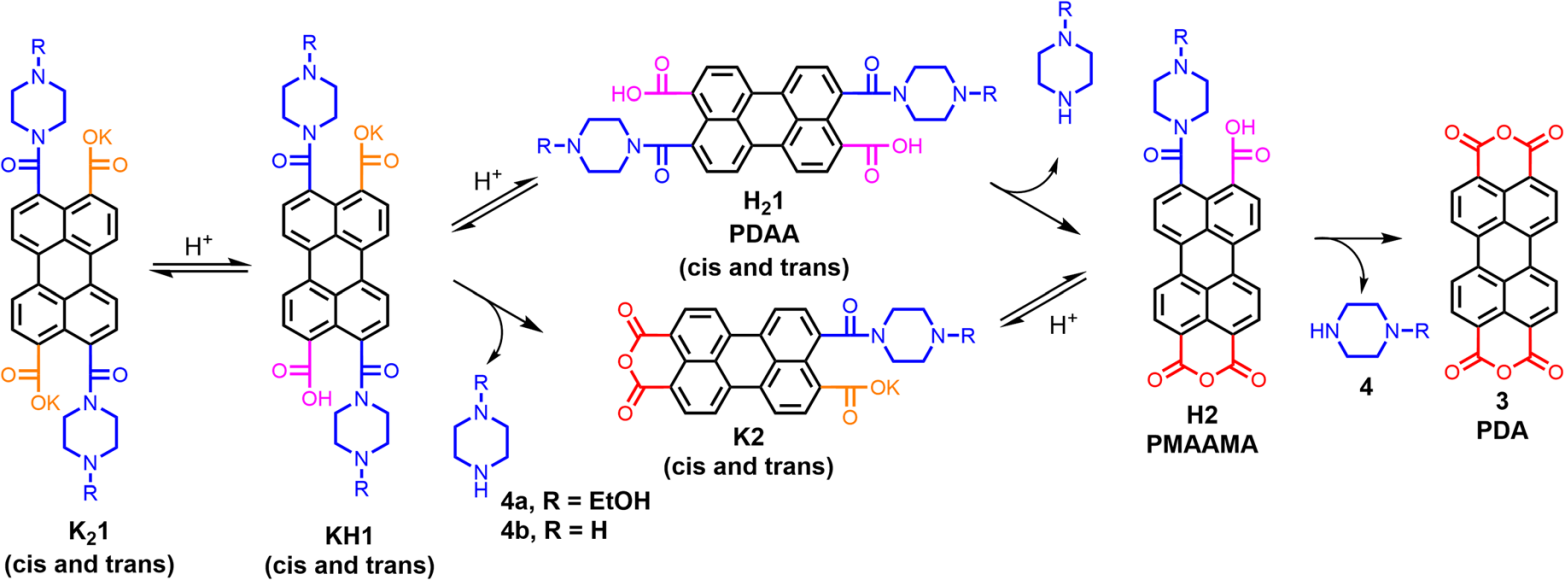
Protonation and hydrolysis of PDAA hydrogelator K_2_1 forming PDA 3*via* two different pathways.

In this work, perylene-3,4,9,10-tetracarboxylic acid diamic acid salts (PDAA salts) are introduced as a novel type of perylene-based hydrogelator. The gelation of the piperazine-derived PDAA salts K_2_1a and K_2_1b will be investigated, and a two-steps gelation mechanism, in which protonation of the amic acid salts is followed by amic acid hydrolysis yielding perylene-3,4,9,10-tetracarboxylic acid dianhydride 3 (PDA), is proposed, Scheme 1. This work will delve into both the molecular transformations and the consecutive aggregation processes, which result, to the best of our knowledge, in the first reported PDA hydrogels.

## Results and discussion

Amic acid salts are conveniently synthesized by the reaction of amines with anhydrides. When primary amines are used, the formed amic acids are the intermediates in the synthesis of imides, but when secondary amines are employed, amic acid salts are stable against further imidization.^12^ For the synthesis of PDAA salts, as depicted in Scheme S1,† K_2_CO_3_ was used as a base, since the potassium salts K_2_1 are solid materials that are conveniently isolated and characterized.

When subjected to acidic conditions, PDAA salts K_2_1 will revert back to their starting material PDA. We have serendipitously discovered that this reaction induces hydrogel formation from crude reaction mixtures diluted with water. Hydrogels were successfully formed from various PDAA salts by acidification with mineral acid, buffer solutions, or with glucono-δ-lactone (GdL),^13^ a compound that slowly hydrolyses in water while forming a carboxylic acid, as shown by Fig. S2, Scheme S1 and Table S1.†

**Fig. 1:**
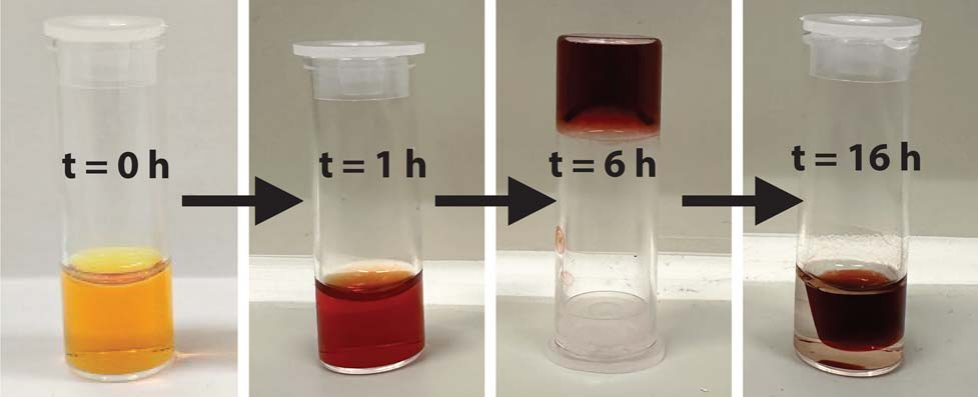
Gelation of 1 mM K_2_1a using 0.05 M glucono-δ-lactone (GdL).

To gain a better understanding of the gelation process, gel formation was investigated using PDAA salts K_2_1a and K_2_1b in water, a solvent in which these compounds are molecularly dissolved.^14^ Gelation was achieved by slowly lowering the pH of K_2_1 solutions using freshly prepared GdL solutions. In Fig. 1, S3, S4, Videos V1 and V2,† gel formation at different concentrations, was monitored. It is clearly visible that upon acidification the colour intensifies and is red-shifted while the green-blue fluorescence disappears. After 6–10 hours, an invertible hydrogel is formed at gelator concentrations ≥0.5 mM, a value that is one order of magnitude lower than the 4 mM critical gel concentration (CGC) reported for phenyl alanine appended PDI,^10*a*^ see Table S2.†

After gel formation, syneresis occurs and the rate and extent of syneresis increases at decreasing gelator concentrations. Below 0.5 mM, samples already shrunk before developing the required mechanical strength for the reversed tube test. We do note, however, that shape-persistent gels, that are stable in solution, were formed at concentrations as low as 0.015 mM, see Fig. S3 and S5.†

Gel formation from K_2_1 is a robust process. Stable hydrogels were formed at elevated temperatures up to 65 °C and both the rate of gel formation and the rate of syneresis were strongly enhanced, see Fig. S6–9.† This demonstrates that elevated temperatures do not interfere with gel formation. Gelation was also successful in a variety of acidic buffers, with pH values between 3 and 5, see Table S3,† and as noted before, hydrogels are formed with a large variety of PDAAs in crude reaction mixtures.

Gel formation from K_2_1 is not a reversible process, as is generally the case for gels formed by lowering the temperature or changing the pH. Gel objects immersed in 0.1 M K_2_CO_3_ solutions slowly dissolved, while forming strongly fluorescent perylene 3,4,9,10-tetracarboxylate 5^4−^ solutions, see Fig. S10.†^15^ Direct formation of PDA gel, by protonation of K_4_5 in a GdL solution was attempted, see Scheme S3.† However, despite the slow and spatially controlled protonation by GdL, precipitation of PDA was observed^16^ instead of gel formation. Apparently, 1-D growth of partially protonated 5 does not occur prior to PDA formation and precipitation.

**Fig. 2:**
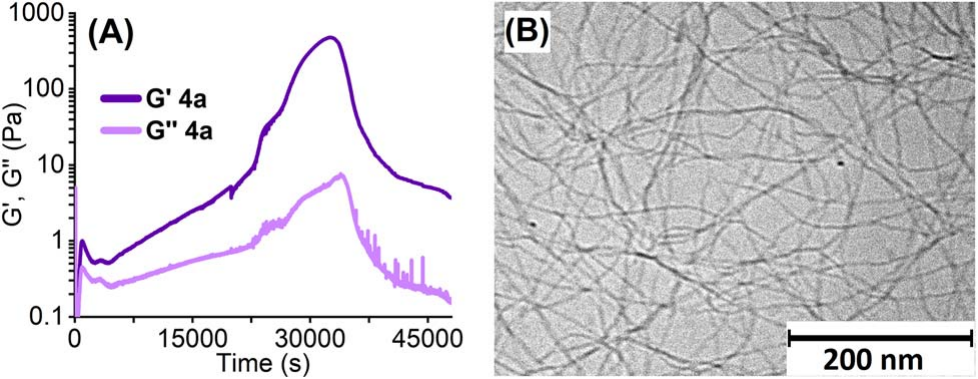
(A) Rheological time-sweeps of *G*′ and *G*′′ (0.5% strain, 1 Hz at 20 °C) of 1 mM aqueous gels of K_2_1a prepared with 0.05 M glucono-δ-lactone (GdL). (B) Cryo-TEM image of the hydrogel formed with K_2_1a (*c* = 1.0 mM) and glucono-δ-lactone (GdL) 0.05 M.

The mechanical properties of hydrogels were examined by oscillatory rheology using 1 mM K_2_1 in 0.05 M GdL solutions. In time-sweep experiments, the storage (*G*′) and loss (*G*′′) moduli were measured over time at 20 °C, see Fig. 2A, S11 and 12.†^17^ In the first few minutes of the gelation process, *G*′ values exceeded *G*′′ values, indicating viscoelastic behaviour and a fast formation of a very weak “amic acid gel”. This was followed, after a short decrease, by a steady increase of the storage and loss moduli to form a strong “PDA gel”.

Gels made from compound K_2_1a exhibit a maximum value for *G*′ of 480 Pa after 9 hours. Afterwards, the storage and loss moduli decreased, as the gel slab detached from the rheometer plates due to syneresis. The gel made with K_2_1b did not detach within the timeframe of the experiment and exhibited a higher value for *G*′ of 670 Pa (Fig. S11†). These *G*′ values are in the same order of magnitude as those of PDI hydrogels reported in the literature, although these PDI hydrogels were characterized at higher concentrations, typically 5–10 mM.^10^ It should be noted, however, that our rheology measurements gave poorly reproducible results, which is partly due to the unpredictable timing of gel detachment. Despite this uncertainty, the viscoelastic behaviour of the formed gels has been clearly established from these measurements.

Gelation of small molecules is usually induced by aggregation processes that form fibres with large aspect ratios that are cross-linked. In Fig. 2B, a cryo-TEM image of a freshly prepared gel from compound K_2_1a made with GdL is depicted. The gel depicted in Fig. 2B consists of long cross-linked fibres and is fairly homogeneous, in contrast to the samples that were prepared by HCl addition (Fig. S13†).^13^ Analysis of the cryo-TEM images revealed that fibres range in diameter from 3.5 nm to 6.5 nm. As the dimensions of a single PDA molecule, perpendicular to the stack direction, are 0.5 by 1.25 nm, these fibres bundle multiple PDA stacks. Providing the presence of PDA molecules only, the number of stacks in a fibre will be around 10–35.^18^

**Fig. 3:**
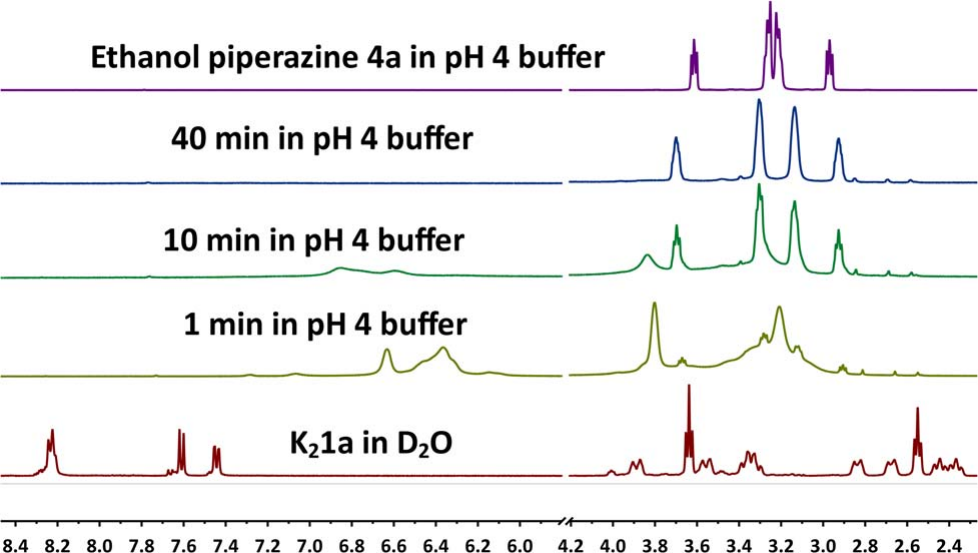
NMR measurement of the gel formation of K_2_1a (80 mM) in deuterated acetic acid buffer (0.67 M) at pH 4.

To investigate the gel formation process on a molecular level, the gelation of K_2_1a in a pH 4 CD_3_COOD/NaOD buffer^19^ was monitored by ^1^H NMR spectroscopy, see Fig. 3, S14 and 15.† Right after acidification, very broad resonances appeared in the aliphatic and aromatic regions of the spectrum. This is indicative of a decreased mobility of the perylene and 1-(2-hydroxyethyl)piperazine groups, likely due to perylene π-stacking. Subsequently, a steady decrease in intensity of the broad aromatic resonance was visible, which is indicative of rigidification of the aggregates. In addition, these resonances shifted to lower field, changed their shape, and disappeared after 30 minutes.

Already after 1 minute, the sharp resonances of 1-(2-hydroxyethyl)piperazine appeared, which steadily increased in magnitude. Beyond 40 minutes, the intensity of these resonances remained constant, while the broad resonances of piperazine moieties attached to aggregates had vanished completely. These observations provide solid proof that 1-(2-hydroxyethyl)piperazine 4a is split off during the gelation process. Also, the instantaneous formation of aggregates by PDAA protonation was demonstrated by the instant broadening of the aromatic and the aliphatic resonances. These aggregates become more rigid and less mobile as the gelation process proceeds, as evidenced by the disappearance of the aromatic resonances.

**Fig. 4:**
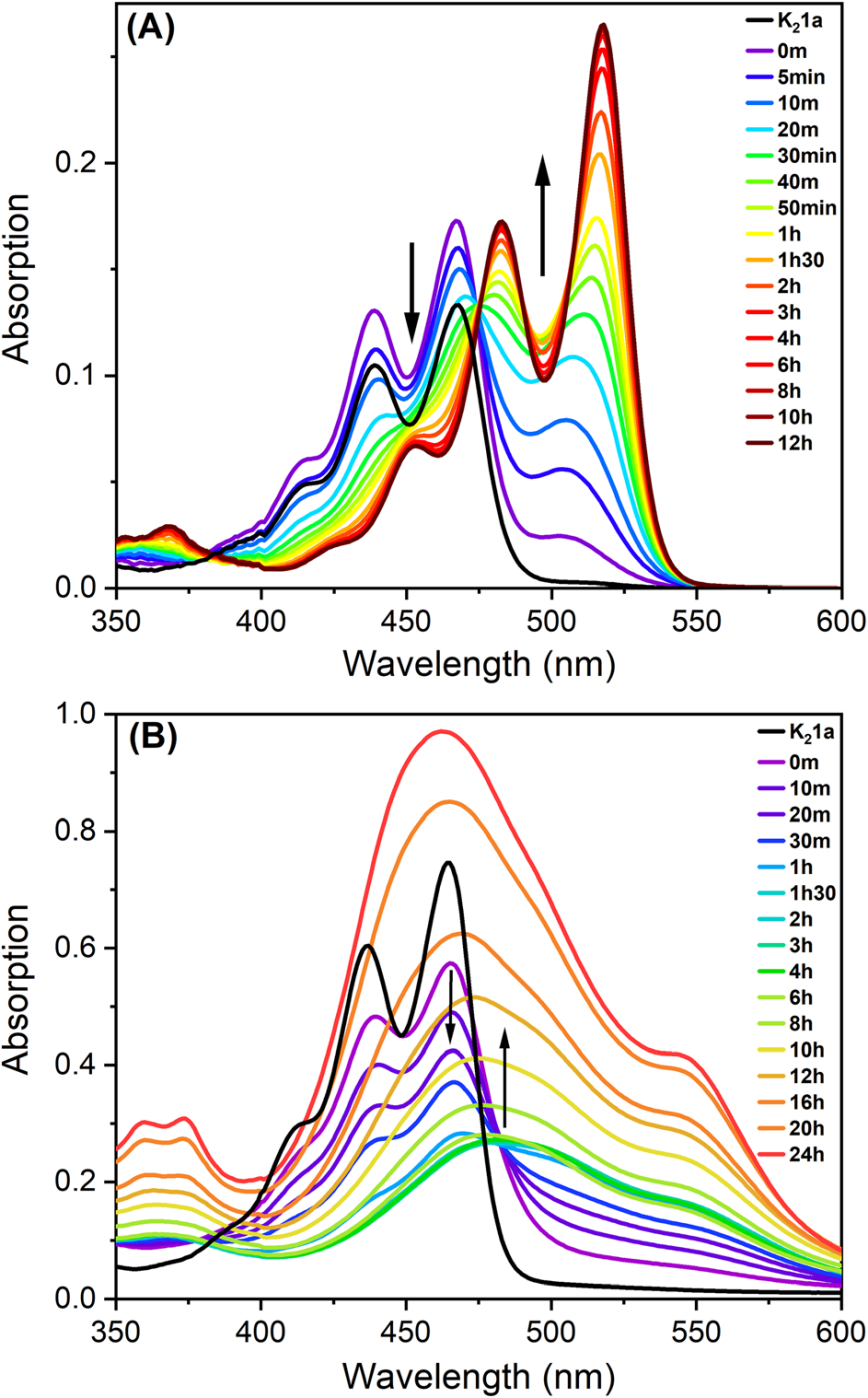
(A) Absorption spectra of the acidification and hydrolysis of K_2_1a in DMF. (B) Absorption spectra of the gel formation of K_2_1a (0.23 mM) with 0.05 M glucono-δ-lactone (GdL).

The formation of the other hydrolysis product PDA 3, which forms the gel fibres, was elucidated by UV-Vis spectroscopy. For this purpose, the reaction of K_2_1a with HCl was monitored in DMF, a solvent in which the starting compounds, intermediates, and reaction products are all soluble, see Fig. 4A. At first, the typical absorption spectrum of amic acid salt K_2_1a can be seen. Upon addition of HCl, amic acid H_2_1a is formed instantaneously, and the fast formation of anhydride H2a is also visible by the broad absorption that emerges at 510 nm. Subsequently, amic acid hydrolysis proceeds, and the final spectrum with absorption maxima at 453, 482, and 518 nm is that of PDA 3.^12,20^ Isosbestic points are not visible in Fig. 4A since PDAA hydrolysis is a two-step process.

Finally, the gelation process of compound K_2_1a was monitored by UV-Vis spectroscopy in water with GdL, as depicted in Fig. 4B. In the first hour, the spectrum of the molecularly dissolved amic acid salt K_2_1a gradually decreased, and a broad, red-shifted and profoundly less intense absorption emerged. The resulting spectrum, with an absorption maximum at 480 nm, remained fairly stable for the following 5 hours. Subsequently, the absorption intensity increased, and the absorption maximum gradually shifted 15 nm to the blue. The composition of the perylene molecules within the aggregates will shift from the protonated PDAA salts, H1a^−^ and H_2_1a*via* compounds 2a^−^ and H2a to PDA 3, Scheme 1. In molecular solutions, these transformations result in pronounced red shifts and strong increases in absorption intensity, while the vibronic structure in the spectra is retained, see Fig. 4A. However, during gel formation, a modest red shift, a strong 70% decrease in absorption intensity, spectral broadening, and a decrease in vibronic structure are observed.

The aforementioned spectral changes observed during the gelation process are very similar to those observed for the acid-induced gel formation of amino acid-appended PDIs^10^ and the aggregation of perylene-3,4,9,10-tetracarboxylic acid tetraesters^21^ (PTEs) or PDIs^7^ in poor solvents. The spectrum of the final hydrogel resembles that of PDI H-aggregates, for which an arrangement of rotationally displaced perylene molecules in π-stacks is proposed.^7,10*c*^ Such a one-dimensional primary structure is also consistent with the fibrilous gel morphology that is revealed by cryo-TEM in Fig. 2B.

In the later stages of the gelation process, beyond 6 hours, the absorption spectra undergo a blue shift, become more narrow, and develop a shoulder at ∼525 nm, see Fig. S16.† These spectral changes coincide with the pronounced increase in the storage moduli in the rheology experiments, after ∼5 hours, which is assigned to the transformation of weak “amic acid” gels to strong PDA gels, Fig. 2a. Likely, the formation of more rigid and highly regular perylene aggregates leads to a stronger exciton coupling within the H-aggregates, which induces the observed blue shifts. Finally, it should be noted that the increased absorption that occurred after 6 hours is largely due to syneresis, as illustrated by Fig. S3 and V1/2.†

For the gel formation of K_2_1, a two-steps protonation–amic acid hydrolysis mechanism has been proposed and validated. Protonation of amic acid salts resulted in the immediate formation of very soft gels, as was proven by rheology experiments. This proton-induced aggregation is followed by hydrolysis of the amic acid functionalities that split off amine 4 and ultimately yields PDA 3, as was proven by ^1^H NMR and UV-Vis measurements, respectively. Along with this chemical transformation, a steady increase in the mechanical properties of the hydrogel and a decreased mobility of the perylene molecules within the aggregates was observed from rheology, NMR, and UV-Vis experiments, respectively. The increase in the mechanical properties is likely a consequence of amic acid hydrolysis, which removes the bulky amide and carboxylic acid groups from the perylene scaffold, that impede efficient π-stacking. PDA 3, that is formed by amic acid hydrolysis, is a planar molecule without such bulky substituents, and this compound is expected to form much stronger and more rigid fibres. Based on these considerations, we anticipate that the increased gel strength is primarily due to changes within the gel fibres,^22^*i.e.* the primary gel structure. The obtained hydrogels are, to the best of our knowledge, the first PDA hydrogels that have been reported. Since we were not able to make PDA gels directly by protonation of the perylene tetracarboxylate salt K_4_5, we anticipate that the stepwise gel formation, *via* soft “amic acid gels”, is a prerequisite for making PDA hydrogels. Also, because PDA is not known to spontaneously form 1-D aggregates.^23^

Although the chemical reactions that induce gel formation have been elucidated, providing a detailed description of the chemical transformations that take place during our experiments is challenging. This is the case because the sequence of the various protonation and (acid-catalysed) hydrolysis steps strongly depends on the mode of pH lowering. In addition, the amide substituents at the different PDAAs will affect these chemical transformations as well since the rates of hydrolysis, the extent of protonation (carboxylic acid p*K*_A_s), all depend on the molecular structure of this substituent. Another uncertainty, with regards to the chemical conversions during gel formation, is whether or not PDA is formed quantitatively. In (DMF) solution this conversion is quantitative, but during gel-formation in water it is conceivable that a small part of the amic acid functionalities are trapped within the gel and remain intact. It should be noted, that the complexity of the gelation makes this process tuneable by variation of both the acidification protocol and the molecular structure of K_2_1. Preliminary experiments have demonstrated that indeed PDA gels with a wide range of properties can be prepared by changing these parameters.

## Conclusions

In conclusion, we present a new class of perylene-based hydrogelators; perylene diamic acid salts K_2_1, which, at sub-millimolar concentration, form highly coloured hydrogels upon decreasing the pH. For gel formation, a two-steps protonation–hydrolysis mechanism is proposed and validated. In this mechanism, a fast protonation takes place, which forms insoluble amic acids that form weak hydrogels. This protonation is followed by a slow hydrolysis, which eventually results in the formation of PDA 3, a compound that forms much stronger gels. The resulting hydrogels are the first reported PDA hydrogels, and these PDA-hydrogels exhibit respectable mechanical properties, with *G*′ values up to 600 Pa @ 1 mM and undergo substantial syneresis. The formation of PDA hydrogels is a robust and widely applicable process that works with multiple PDAA salts under various gelation conditions.

Further research will focus on elucidating the two-step gelation mechanism and the subsequent syneresis in more detail. With this knowledge it should be possible to make gels with a wide range of properties by either changing the molecular structure of the gelator or the mode of acidification. In terms of application, the low toxicity of PDA and the amines that are split off during hydrolysis, along with the potential photoresponsivity and electrical conductivity of the gels, offer opportunities for applications that are currently under investigation.

## Data availability

The data supporting this article have been included as part of the ESI.†

## Author contributions

M. K, P. vd B., N. S. and M. C. K. conducted most experiments. W. F. J. supervised the research, while the manuscript was written by M. C. K. and W. F. J.

## Conflicts of interest

There are no conflicts to declare.

## Supplementary Material

RA-015-D5RA00372E-s001

RA-015-D5RA00372E-s002

RA-015-D5RA00372E-s003
