# Supplementary material for: Perylene dianhydride hydrogels obtained from accessible perylene diamic acid salts by a versatile protonation–hydrolysis mechanism
Source: RSC Adv. 2025 Apr 2;15(13):10183–7. doi: 10.1039/d5ra00372e (PMC11962865; doi:10.1039/d5ra00372e)
Supplement: RA-015-D5RA00372E-s001 [file RA-015-D5RA00372E-s001.pdf]

Supplementary Information (SI) for RSC Advances.
This journal is © The Royal Society of Chemistry 2025

Supplementary Material for

Perylene dianhydride hydrogels obtained from accessible perylene diamic acid precursors by a versatile protonation-hydrolysis mechanism

Markus C. Kwakernaak,[a,b] Marijn Koel,[a] Peter J. L. van den Berg,[a] Nicolaas Strik,[a] and Wolter F. Jager*[a]

a: Department of Chemical Engineering, Delft University of Technology, Van der Maasweg 9, 2629 HZ Delft, The Netherlands. b: Department of Radiation Science and Technology, Delft University of Technology, Mekelweg 15, 2629 JB Delft, The Netherlands.

W.F.Jager@tudelft.nl

Table of Contents 2

Instrumentation and characterization of gels and fel formation..3
Synthetic procedures 4
Gel formation in water 5
Gel formation from crude reaction mixtures...7
Schemes, Figures and Tables. 8
UV-Vis and NMR Spectra……………………………………………………………… 18

Instrumentation

NMR spectra were recorded with an Agilent 400-MR DD2 (400 MHz) pulsed Fourier transform NMR spectrometer in MeOH-D4 or D_2O at room temperature. Chemical shifts are given in ppm and J values in Hz. Absorption measurements were performed using a Perkin Elmer Lambda 365 UV−Vis spectrophotometer. Rheology measurements were taken using a TA Instruments Discovery HR-3 Hybrid Rheometer. TEM images were acquired using a JEOL JEM1400 transmission electron microscope operating at 120 kV.

Characterization of gels and gel formation

Rheology measurements gel formation
Stock solutions of 2 mM PDAA salts $\mathbf{K_21}$ in demi-water were prepared. 1 mL of this stock was mixed with 1 mL of freshly prepared 0.1 M GdL solution. This was poured on the plate of the rheometer. Subsequently the storage and loss moduli were measured on oscillation time setting with a gap size of 550 μm, 0.5% strain rate and 1 Hz frequency at 20°C. These conditions, apart from the gelator concentration are identical to those used for amino acid PDIs in references 10a and 10c. Measurements were done with parallel flat plates.

NMR gel formation
A solution of $\mathbf{K_21a}$ was prepared by dissolving 53 mg of $\mathbf{K_21a}$ in 0.45 ml D_2O. An acetate buffer was prepared by dissolving 35 μL deuterated acetic acid and 16.7 μL 30 wt.% NaOD solution in 0.45 ml D_2O. The pH was adjusted to pH=4, as determined by pH paper, by adding NaOD. The solution of $\mathbf{K_21a}$ and the acetate buffer were mixed in a 1:1 ratio and transferred to an NMR tube, after which the sample was measured over 15 hours at 20°C.

UV-Vis gel formation
For the time dependent UV-VIS gel experiment, a stock solution of $\mathbf{K_21a}$ was prepared by dissolving 8.56±0.01 mg of $\mathbf{K_21a}$ in 50 mL H_2O. A 0.1 M GdL solution was prepared and immediately mixed with the $\mathbf{K_21a}$ stock at a 1:1 ratio. The mixture was transferred to a 1mm cuvette and measured over 24 hours 25°C. A reference spectrum (for t=0) was obtained by dissolving 0.15 mL $\mathbf{K_21a}$ stock in 2.85 mL H_2O in a 1 cm cuvette without the addition of GdL.

UV-Vis Hydrolysis in DMF
For the molecular hydrolysis experiment, a stock solution 0.1 mg/mL of $\mathbf{K_21a}$ is prepared in DMF. 200 μL of this solution is further diluted with 2.7 mL DMF. The mixture was transferred to a 1 cm pathlength cuvette and measured at 25°C. After this initial measurement, 50 μL of 3 M HCl is added and very quickly mixed and measured over 24 hours.

Cryo-TEM imaging
Hydrogels made with compound $\mathbf{K_21a}$ were imaged by cryo-TEM. A 2.0 mM stock solution of $\mathbf{K_21a}$ in demineralized water was made and 1.0 mL of this solution was mixed with 1.0 mL of freshly prepared 0.1 M GdL. After 6 hours this had formed a gel, 3 μL was pipetted from the solution, which was blotted for 2s before being plunged into liquid ethane to freeze rapidly. This was then examined using the TEM microscope operating at 120 kV. From 2.0 mL of the same $\mathbf{K_21a}$ solution a gel was made by adding a 200 μL of 1 M HCl solution. The gel, formed in 1 hour, was processed in the same fashion as the “GdL gel”.

Synthetic procedures

Perylene-3,4,9,10-tetracarboxylic acid tetrapotassium tetracarboxylate (**$K_4$5**) was synthesized as described in the literature.[1] All other reagents were supplied by Sigma Aldrich and used as received, unless stated otherwise.

*Perylene-3,4,9,10-tetracarboxylic acid-3,9-di(N-piperazyl-N'-2'-hydroxyethyl)amide-4,10-dipotassium dicarboxylate **$K_2$1a** and Perylene-3,4,9,10-tetracarboxylic acid-3,9-di(N-piperazyl)amide-4,10-dipotassium dicarboxylate **$K_2$1b***

To a 50 mL round bottom flask, DMSO (20 mL), PDA **3** (1.00 g, 2.55 mmol), K_2CO_3 (0.70 g, 5.10 mmol) and the appropriate amine **4** (10.2 mmol, 4 eq) were added. The resulting slurry is stirred for 2 hours at 60 ^{o}C. The mixture is left to cool to room temperature and then poured into acetone (200 mL) to precipitate the product. The solid is filtered off and dissolved in methanol (10 mL) and filtered again. The precipitation in acetone (200 mL) is repeated. The potassium amic acid salts **$K_2$1** were isolated as yellow/orange solids. Isolated yields were 1.21g g (65%) of **$K_2$1a** and 0.78g (48%) of **$K_2$1b**.

^{1}H NMR **$K_2$1a** (CD_3OD, 400 MHz): 8.36 (4H, m), 7.80 (2H, J= 8Hz, d), 7.49 (2H, J= 8Hz, d), 4.01 (2H, J= 12Hz, d), 3.67 (4H, J= 6Hz, t), 3.63 (2H, m), 3.50 (4H, m), 2.91 (2H, J= 12Hz, d), 2.77 (2H, J= 12Hz, d), 2.59 (4H, J= 6Hz, t), 2.38 (4H, m).
^{13}C NMR (CD_3OD, 101 MHz): 175.64, 175.61, 171.33, 171.30, 139.49, 139.13, 133.77, 133,.40, 132.10, 131.03, 128.91, 128.35, 127.61, 127.26, 127.16, 121.16, 120.90, 120.13, 59.72, 58.47, 52.68, 52.51, 41.21.
UV-Vis (water): λ_{max} (ε)= 414 (shoulder), 437 (27000), 464 (33500).

^{1}H NMR **$K_2$1b** (CD_3OD, 400 MHz): 8.37 (4H, m), 7.81 (2H, J= 8Hz, d), 7.51 (2H, J= 8Hz, d), 3.96 (2H, J= 12Hz, d), 3.59 (2H, m), 3.46 (4H, m), 3.10 (2H, J= 12Hz, d), 2.95 (2H, J= 12Hz, d), 2.78 (4H, m).
^{13}C NMR (CD_3OD, 101 MHz): 175.60, 175.58, 171.50, 171.48, 139.45, 139.09, 133.76, 133,.40, 132.29, 132.10, 131.05, 130.84, 128.94, 128.38, 127.63, 127.52, 127.24, 127.14, 121.15, 120.90, 120.13, 119.89. 44.66, 44.47, 29.22.
UV-Vis (water): λ_{max} (ε)= 414 (shoulder), 436 (27000), 464 (33500).

Gel formation in water

Video V1. Gelation of a 1.0 mM solution of **$K_2$1a** with freshly prepared 0.05 M GdL solution in demi water in a 4 mL vial. The duration of the video is 1 hour.

Video V2. Gelation of a 1.0 mM solution of **$K_2$1a** with freshly prepared 0.05 M GdL solution in demi water in a 4 mL vial. The duration of the video is 18 hours.

Forming Hydrogels with GdL

Solutions of **$K_2$1** with twice the desired hydrogel concentration ($5*10^{-7}$-$5*10^{-3}$M, see Figure S3) were prepared in distilled water. A 0.1 mM glucono-δ-lactone (GdL) in water was prepared and after full GdL dissolution, the two solutions were mixed in equal amounts (1 mL) immediately. This yielded fluorescent solutions that turned into non-fluorescent red solutions within an hour. Approximately 6 hours (**$K_2$1a**) and 10 hours (**$K_2$1b**) into the experiment, the red solutions gelated to the point that a gel inversion was possible, Figure S4. Critical gel concentrations (CGCs) were 1.0 and 0.5 mM for **$K_2$1a** and **$K_2$1b**, respectively. Samples were prepared in duplo; one sample was used for the inversion experiment, the other sample was left undisturbed.

Mechanical properties versus gelator concentration

For samples with gelator concentrations below 0.5 mM, inversion was not possible because syneresis occurred before the gel had developed the modulus required for inversion (~ 10 Pa). For gel objects down to a concentration of 0.015 mM, the cylindrical gel plug remained in shape when the vial was carefully rotated, Figure S5, but collapsed when shaken or when the liquid layer was removed. These gel plugs, however have shrunk to ~20% of their original volume and thus have a significantly higher effective gelator concentration

Forming hydrogels with HCl and different buffers.

Hydrogels were formed by:

- adding acid (HCl, 0.1 and 1M) to a stirred solution of **$K_2$1** (0.1-2.5 mM) in demi water
- mixing a solution of **$K_2$1** (0.2-5 mM) in demi water with an equivalent volume of a buffer (generally 0.2 M)

Gelation times were determined by the inverted test tube method.

Forming hydrogels with GdL at elevated temperatures

To a series of six 5mL aspirin tubes were added 1 mL of a 2.0 mM solution of **$K_2$1a** in demi water. Two Tubes were kept at room temperature (20°C) and the remaining four tubes were pairwise inserted into heating plates that was set at 50 and 60°C, respectively. Subsequently 1 mL of a freshly prepared 0.1 M GdL solution was added to the tubes and the gelation process was monitored over the next hours. Half of the tubes were used for determining the gelation time by the inverted test tube method, while the others were left undisturbed. Gelation occurred in 6 hours, ca 1 hour and well withing 1 hour, respectively. In a second experiment two gel samples (1 mM **$K_2$1a**, prepared with 0.05 M GdL in 6 hours) were aged at 50°C and 20°C, respectively. Results are shown in Figures S6 and S7
The same experiments were performed using a 2 mM **$K_2$1a** solution and a 0.2 M Ascorbic acid pH= 4 buffer solution in equals amounts (1 mL). Gels were made at room temperature (20°C) and at 50 and 65°C. Gelation times (inverted test tube) were 90, <30 and 10 minutes at these temperatures, respectively, Figures S8 and S9. For these samples syneresis at 50 and 65°C was much faster than at room temperature.

Forming Hydrogels from $K_4$5 with GdL
Solutions of **$K_4$5** with twice the desired hydrogel concentration ($5*10^{-7}$-$5*10^{-3}$M), as in Figure S3) and 0.1 mM glucono-δ-lactone (GdL) were prepared in distilled water. After full GdL dissolution, the two solutions were mixed in equal amounts (1 mL) immediately. This yielded fluorescent solutions that became entirely colorless and in which a red precipitate was formed in a matter of 6-8 hours.

Dissolving gel in K_2CO_3
Gel objects made with gelator concentrations of 1, 0.5 and 0.25 mM **$K_2$1a** were made in 5 mL aspirin tubes with 0.05M GdL 40 hours in advance. The colourless liquid was pipetted away from the shrunken gel objects and was replaced by 0.1 M K_2CO_3 solution. This procedure was repeated and the gel objects were immersed in 4 mL 0.1 K_2CO_3 solution. The objects slowly dissolved, at a similar pace as PDA powder received from the manufacturer.[2] In ~6 hours the gel particles were fully disintegrated and formed a precipitate at the bottom of the aspirin tube. The gels were fully dissolved within 24 hours, Figure S10.

Gel formation from crude reaction mixtures

For the preparation of the DBU salts of amic acids **$H_2$1a- $H_2$1d**, a stock solution consisting of 10 mL of DMF and 760 mg, 5 mmol DBU was prepared. PDA 100 mg, 0.25 mmol was suspended in 2.1 mL stock solution containing 1.0 mmol DBU. To this solution 1.0 mmol of amine **4** was added. The reaction mixture was stirred overnight at room temperature.
When using the amines **4e** (an amino acid) and **4f** (used as an HCl salt) a stock solution with 1.14 g, 7.5 mmol DBU was used.

For the gelation experiments 18 mL of demi water was added to the reaction mixture containing the hydrogelator **1**$^{2-}$. From this solution 3.0 mL was added to a 5 mL aspirin tube. Depending on the amine used for preparing **1**, 0.9 (with **4a**, **4b**), 0.7 mL (with **4c** and **4d**) or 0.5 mL (**4e** and **4f**) of a 0.5 M freshly prepared GdL solution was added. In all cases an excess of acid is formed upon complete GdL hydrolysis. The PDA concentration in the final gels are ~10 mM.

Gelation times were determined by the inverted test tube method. The degree of shrinkage was determined by removing the expelled water out of the aspirin tube and determining the mass of the gel plug. Samples were prepared in duplo; one sample was used for the inversion experiment, the other sample was undisturbed and used to determine the degree of shrinkage. Results are displayed in Table S1.

Alternatively crude reaction mixtures were prepared, as described above, diluted with 9 volumes of water and acidified with 0.1-1.0 M HCl until the green-blue fluorescence has disappeared, Figures S1-2.

Schemes, Figures and Tables.

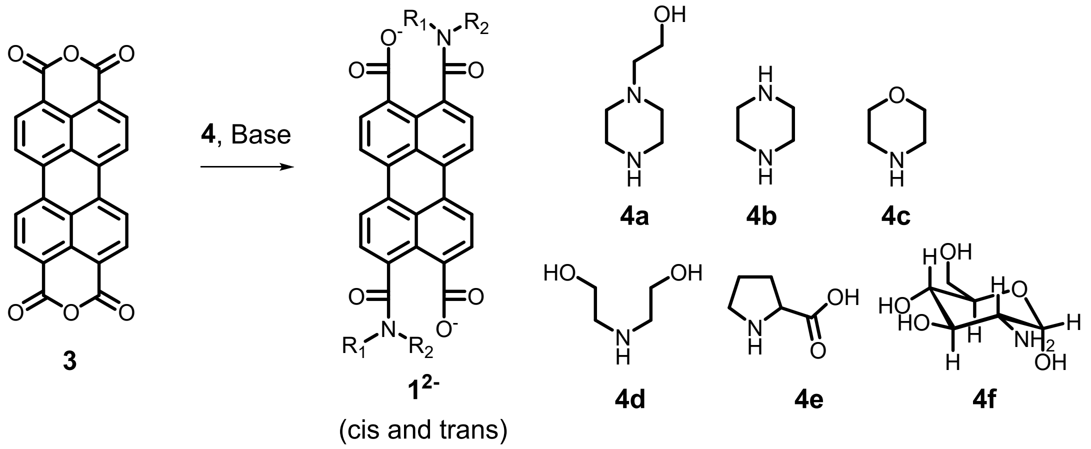

Scheme S1. Synthesis and molecular structure of the amic acid salts ($\mathbf{1}^{2-}$). For the synthesis of $\mathbf{K_21}$, a compounds that are isolated in pure form, K_2CO_3 was used as the base, DMSO is the solvent and the reaction was run for 2 hours at 60°C. For the synthesis of DBU salts of $\mathbf{1}^{2-}$, DBU and DMF are used as base and solvent, respectively, and the reaction was run at room temperature for 24 hours. The resulting reaction mixtures are used for gelation experiments.

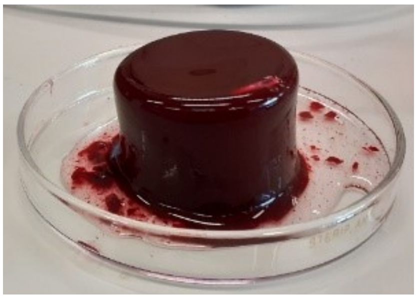

Figure S1. Gel formed from a crude reaction mixture (200 mg PDA, 4 mL DMF), diluted with 36mL water and acidified using 1M HCl under stirring. The hydrogel contains 12.5 mM gelator (DBU salt of **1d**, 0.85% w/w), excess amine and DBU and ~10% DMF. The stirring bar is visible on the top right side of the gel.

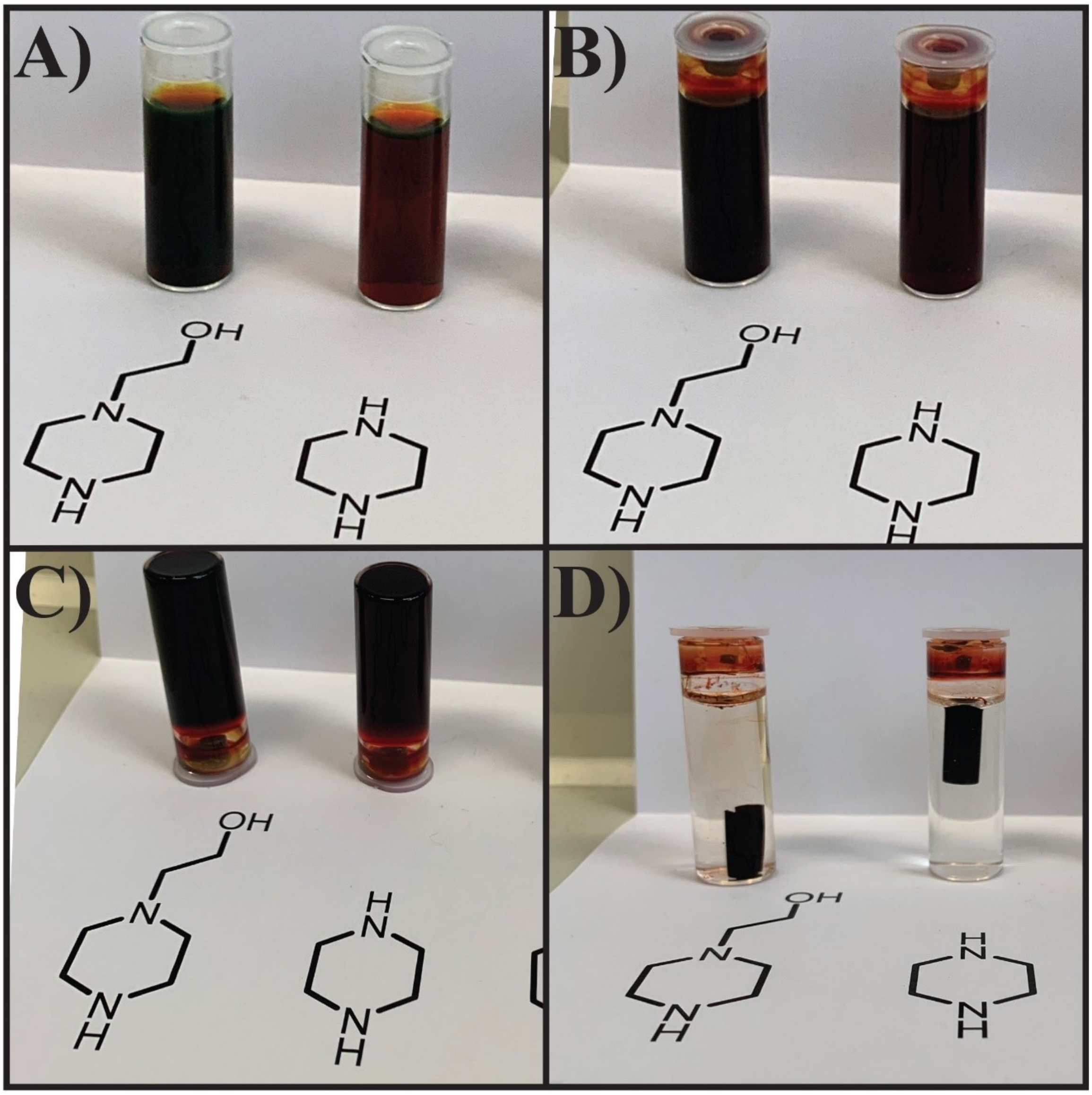

Figure S2. Gelation of crude amic acid reaction mixtures with 0.1 M HCl. The hydrogels contain 12.5 mM gelator (DBU salts of **1a** or **1b**, 0.5-1% w/w), excess amine and DBU and ~10% DMF. **A)** Solutions before adding HCl; **B)** Solutions after HCl addition and mixing; **C)** Freshly formed gels, 25 minutes after acidification; **D)** Shrunken gels, 20h after acidification.

Table S1. Gelation time (inverted test tube) and shrinkage percentage (after 7 days) of amic acid hydrogels (~10 mM, obtained directly from reaction mixtures. Acidification is achieved by adding glucono-δ-lactone (GdL) in excess.

Gelator	**1a**	**1b**	**1c**	**1d**	**1e**	**1f**
Gelation time(min)	330	390	75	90	180	35
Shrinkage (%)	~82	~80	~93	~50	~96	~75

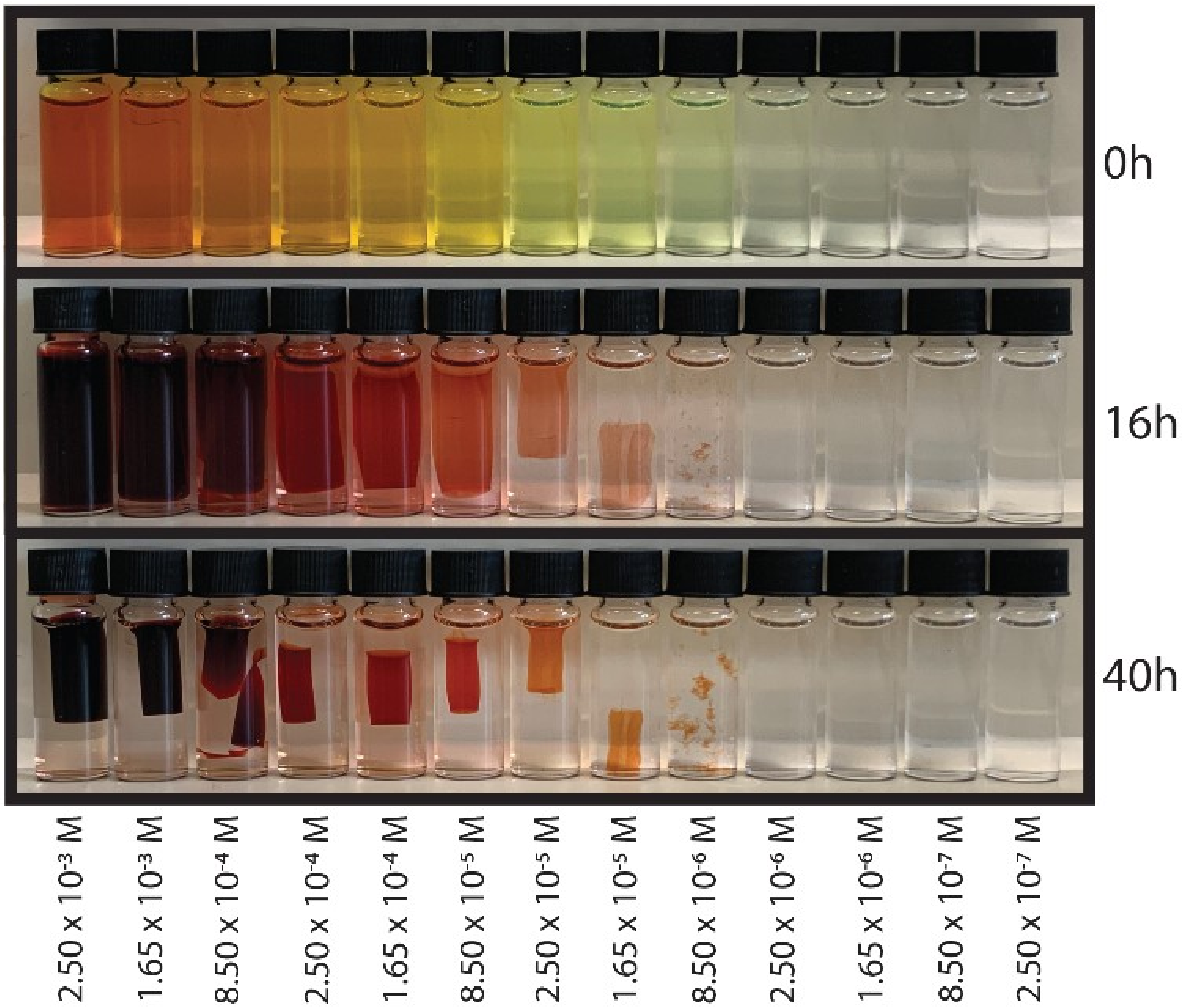

Figure S3. Gelation in 4 mL vials of **$K_2$1a** in water using 0.05 M glucono-δ-lactone (GdL) for acidification.

Table S2. Data obtained from the gelation of 1.0 mM **$K_2$4** in 0.05 mM GdL. [a]: inverted test tube, CGL= critical gel concentration.

Gelator	CGC[a]	Gelation Time[a]	G'_{max} Pa	G''_{max} Pa
**$K_2$4a**	1.0 mM	6h	480	7.5
**$K_2$4b**	0.5 mM	10h	670	6.5

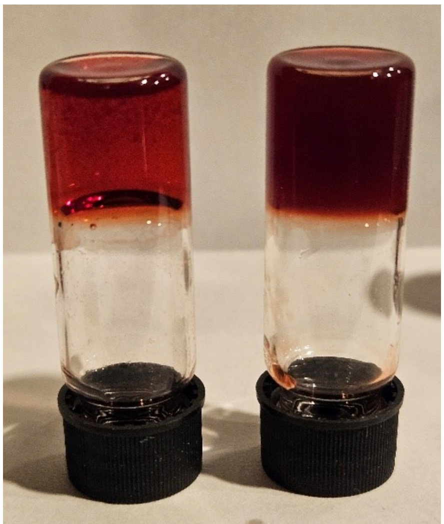

Figure S4. Samples of **$K_2$1b** in 4 mL vials after gelation in water for 10 hours, using 0.05 M glucono-δ-lactone (GdL) at gelator concentrations of 0.5mM (left) and 1.0 mM (right).

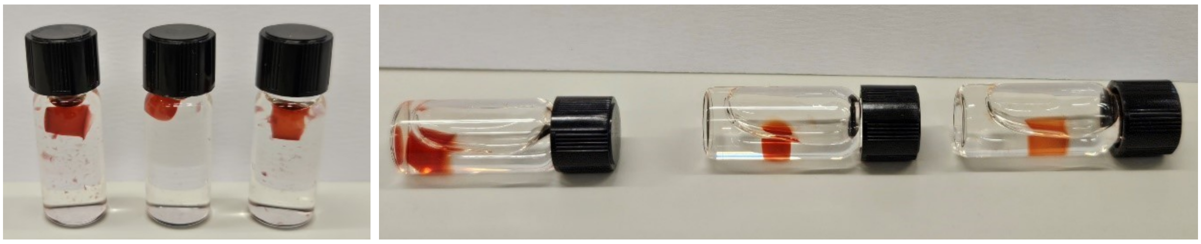

Figure S5. Shape-persistant samples of **$K_2$1b** after gelation in water using 0.05 M glucono-δ-lactone (GdL) at gelator concentrations of 0.1, 0.05 and 0.025mM (left to right). The cylindrical gel objects retained their shape after the vial was carefully rotated.

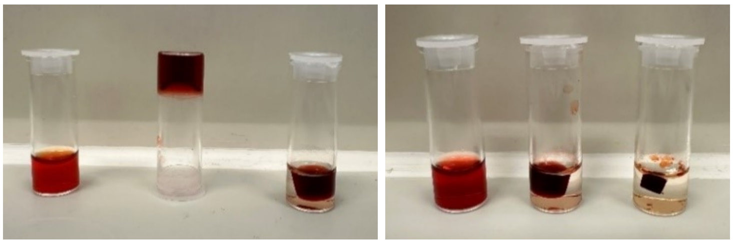

Figure S6. Gelation of 1.0 mM **$K_2$1a** in water using 0.05 M glucono-δ-lactone (GdL) for acidification, performed at room temperature (~20°C, left tube), 50°C middle tube) and 60°C right tube). Photos were taken after 1 hour (left) and 2 hours (right).

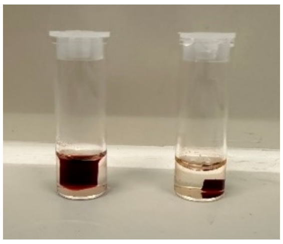

Figure S7. Syneresis of hydrogels (1.0 mM **$K_2$1a** in water, 0.05 M glucono-δ-lactone (GdL)) prepared in 6 hours. Samples were left at room temperature (~20°C, left tube) and 50°C (right tube) for 16 hours.

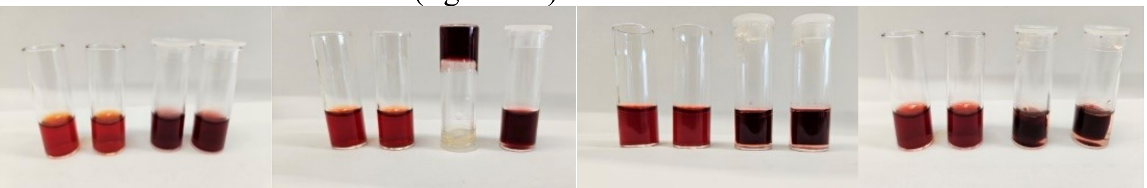

Figure S8. Gelation of 1.0 mM $\mathbf{K_21a}$ in 0.1 M ascorbic acid pH=4 buffer, performed at room temperature (~20°C, left tubes) and at 50°C (right tubes). Photos were taken after 15 minutes, 30 minutes, 75 minutes and 2 hours.

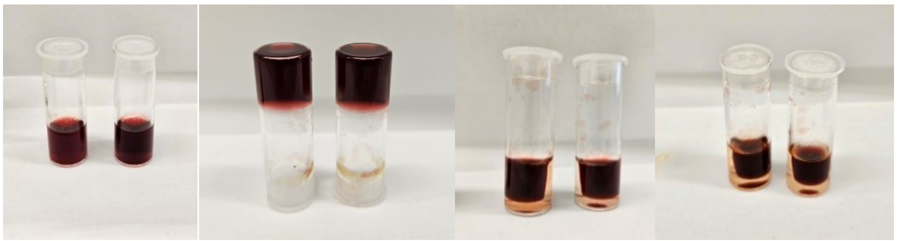

Figure S9. Gelation of 1.0 mM $\mathbf{K_21a}$ in water, using 0.1 M ascorbic acid pH=4 buffer, performed at 65°C. Photos are taken at 0, 10, 30 and 45 minutes.

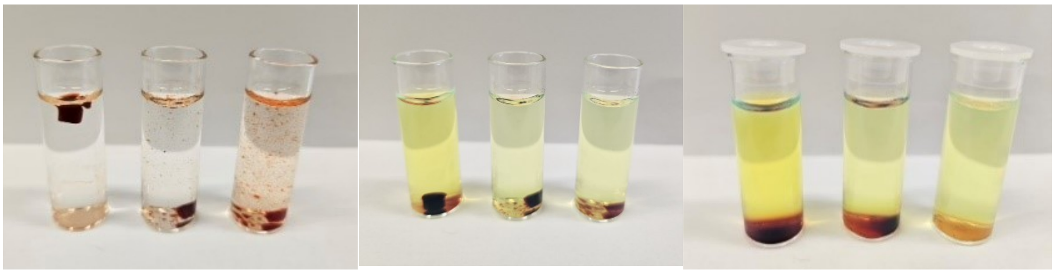

Start 3 hours 6 hours

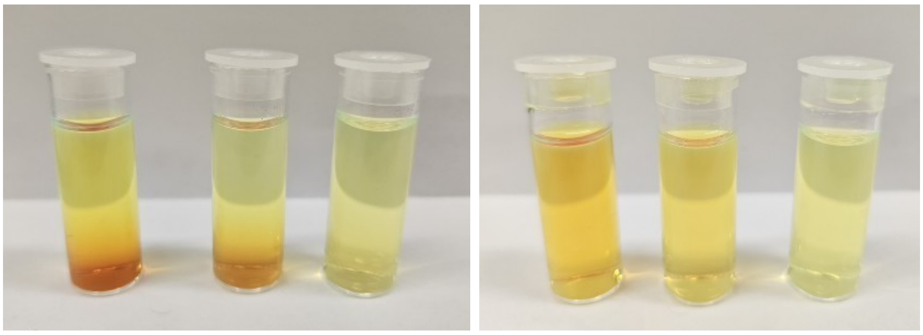

24 hours (undisturbed) 24 hours (shaken)

Figure S10. Dissolution of gel objects made from 1.0, 0.5 and 0.25 mM $\mathbf{K_21a}$ solutions (left to right) with GdL in 0.1 M K_2CO_3 solution, as a function of time.

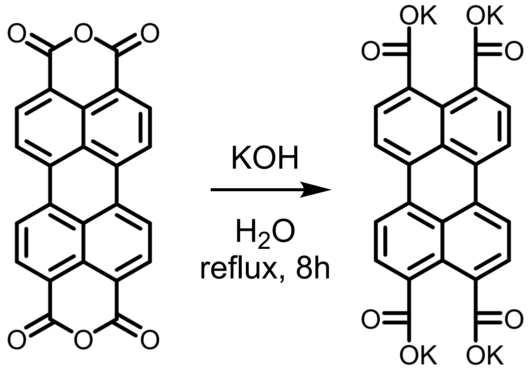

3 $\mathbf{K_45}$

Scheme S2. Synthesis of **K$_4$5**.

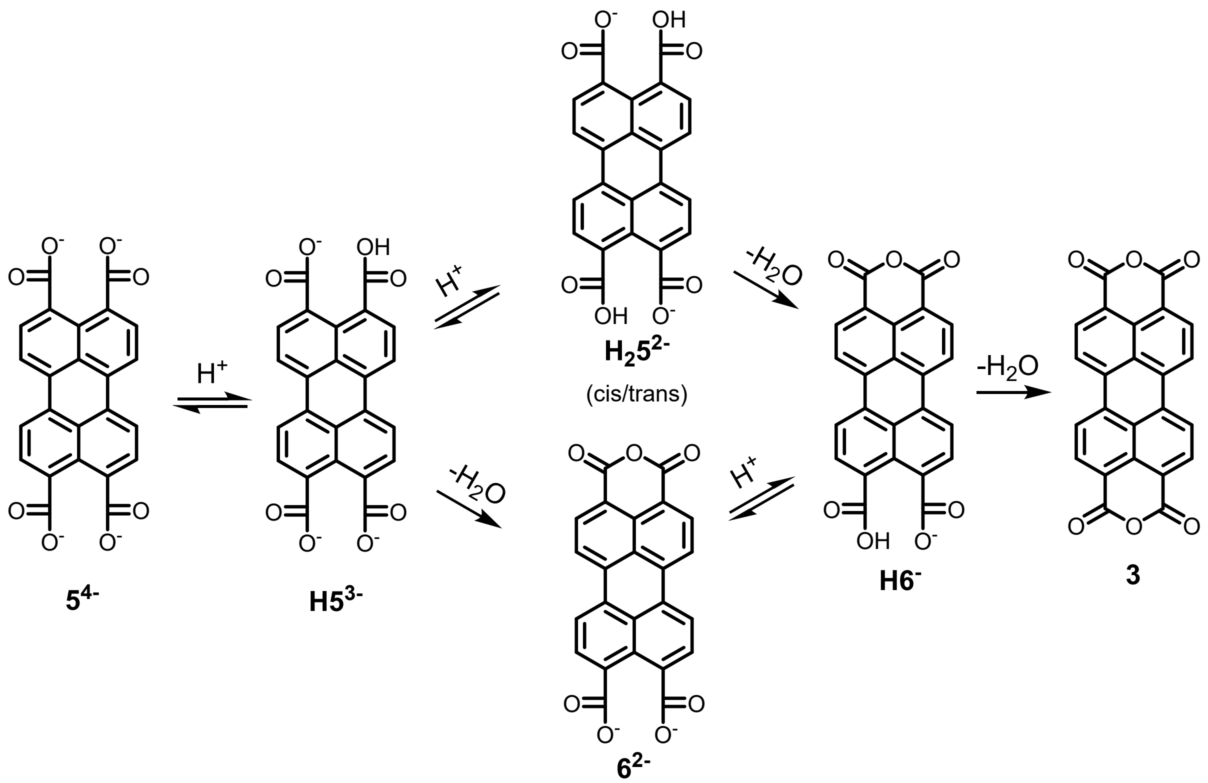

Scheme S3. Pathways for the protonation of **K$_4$5** with GdL.

Table S3. Properties of gels obtained from **K$_2$1a** and **K$_2$1b** in water using various methods to decrease the pH.

Acid/Buffer	pH	Observations	Remarks
HCl	≤ 1	Upon addition of HCl, localized precipitates may form, which redissolves. Precipitation is more pronounced when adding more concentrated HCl (1 M versus 0.1 M HCl) and when working in small volumes, Gels form in 5-10 minutes<br>Gels exhibit fast syneresis, 0.25-1h after HCl addition.	Capable of forming stable hydrogels in large(r) volumes using dilute HCl. Syneresis is fast
glucono-δ-lactone (GdL)	7 → < 3	Upon addition of GdL, samples remain liquid allowing for transfer by pipette. Reproducable formation of hydrogels at GdL concentrations ranging from 0.5-0.05 M. Gels form after 1-10 hours depending on the GdL concentration.<br>Syneresis of gels occurred at an intermediate times between those of HCl (fast) and ascorbic acid buffer (slow).	Capable of forming high quality hydrogels. The rate of syneresis is intermediate
Citric acid	3	Forms hydrogels at buffer concentrations < 0.2 M, while precipitates are formed at higher buffer concentrations. Does not form hydrogels from **K$_2$1b**. Gel formation is very fast and occurs 0.1-0.5 hours after addition of buffer.<br>Syneresis was not monitored.	Capable of forming hydrogels from **K$_2$1a** only
Ascorbic acid	4	Forms hydrogels at buffer concentrations up to 1 M. Stable gels are formed in a highly reproducible fashion**.** Gels form 0.25-2 hours after addition to the buffer.<br>Syneresis is slower than for the GdL gels. Higher concentrations of buffer result in faster syneresis.	Capable of forming high quality long-lived hydrogels with slow syneresis.
Acetic acid	5	Forms gels at acetate buffer concentrations < 0.3 M, while precipitation occurs at higher buffer concentrations. Gels form 0.5-4 hours after addition to the buffer.<br>Syneresis is slowest of all. At higher buffer concentrations crumbling is observed instead of syneresis.	Capable of forming hydrogels with slow syneresis.
Carbonate	6	No appreciable change in the solution was observed at any concentration of carbonate.<br>Gelation did not occur. The color of the solution (and fluorescence) did not change, indicating most of the PDAA was still dissolved	Incapable of forming hydrogels

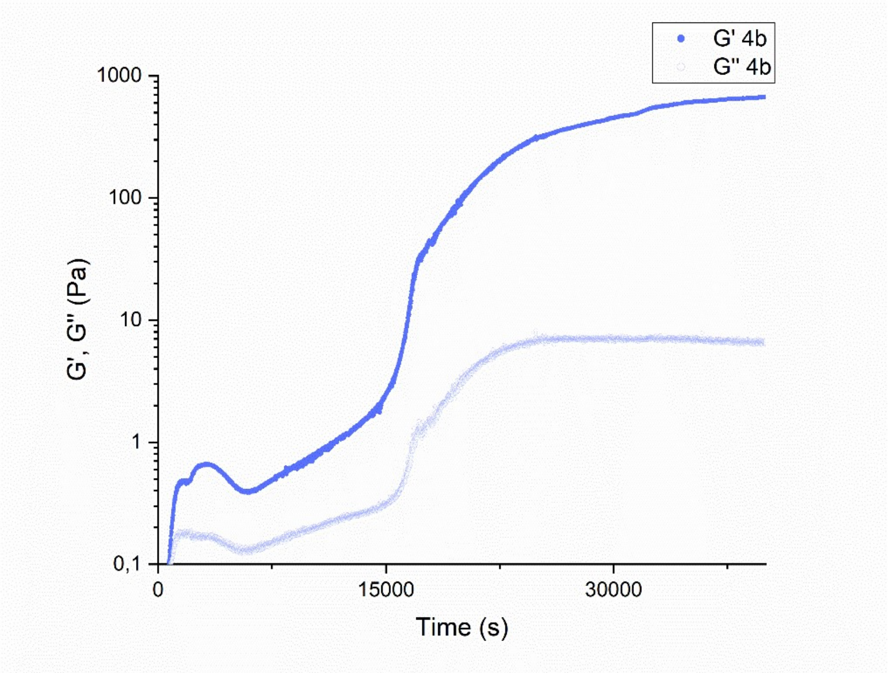

Figure S11. Rheological time-sweeps of G’ and G” of 1 mM aqueous gels of **$K_4$1b** with 0.05 M glucono-δ-lactone (GdL).

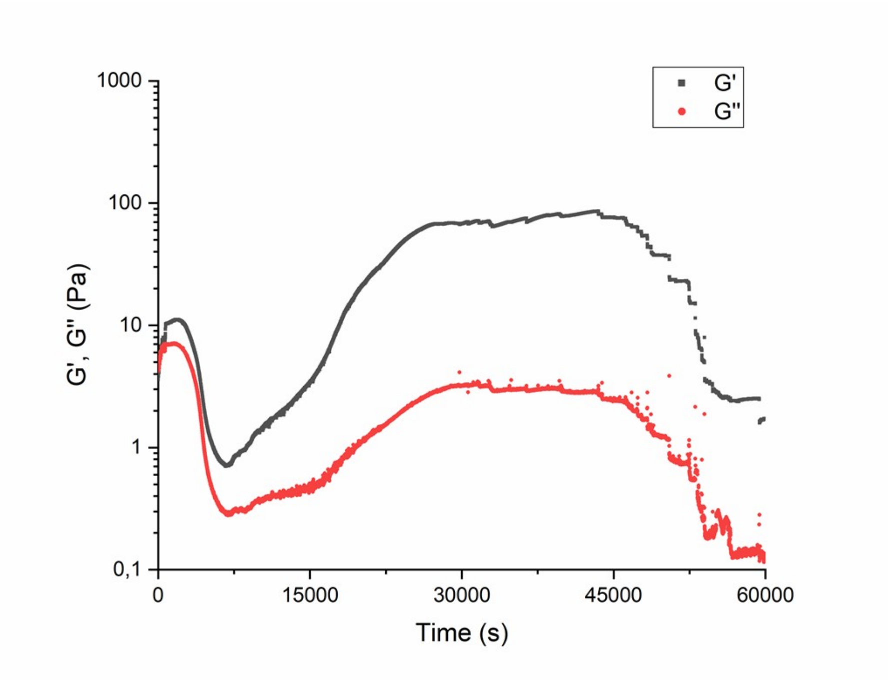

Figure S12. Rheological time-sweeps of G’ and G” of 1 mM aqueous gels of **$K_4$1a** with 0.05 M glucono-δ-lactone (GdL). This is one of the repeated rheology measurements.

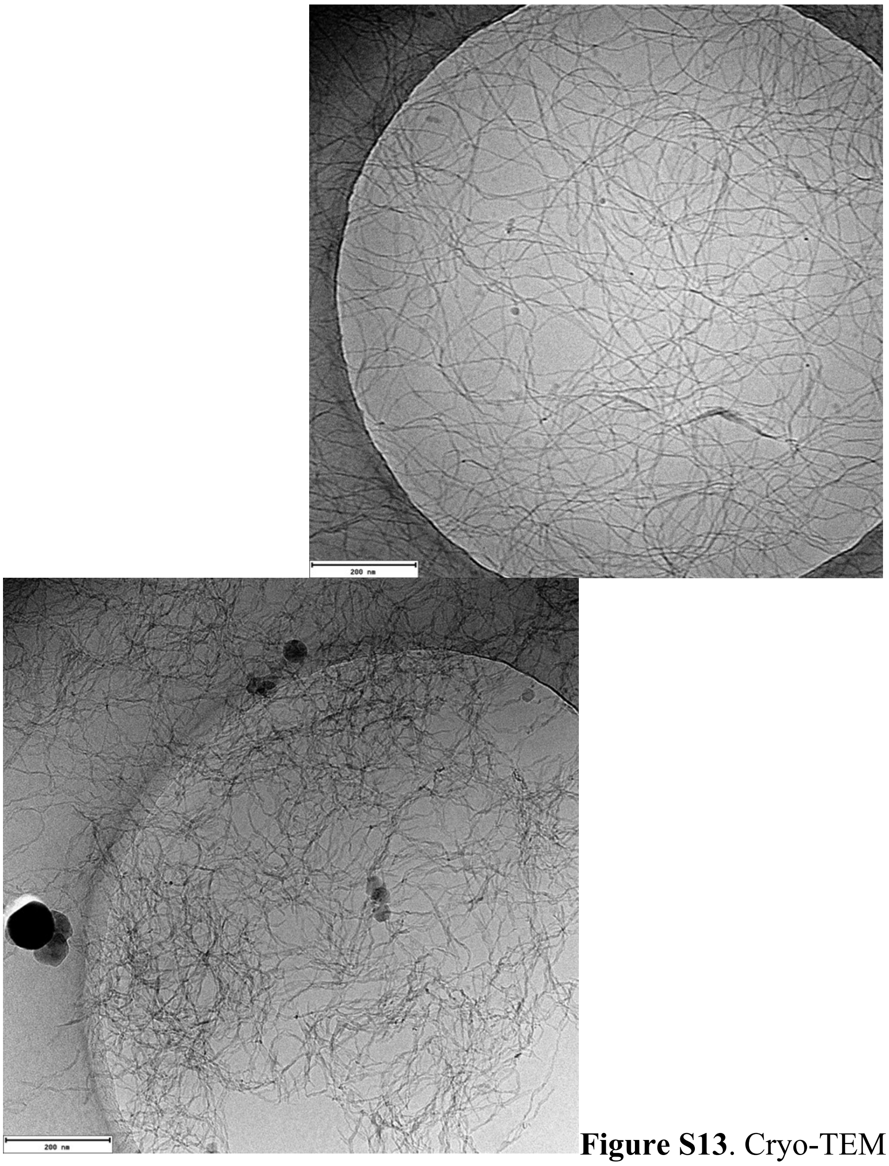

Figure S13. Cryo-TEM images of the hydrogel formed with **$K_2$1a** (c= 0.5 mM) and glucono-δ-lactone (GdL) 0.05 M (left) or 0.1 M HCl (right).

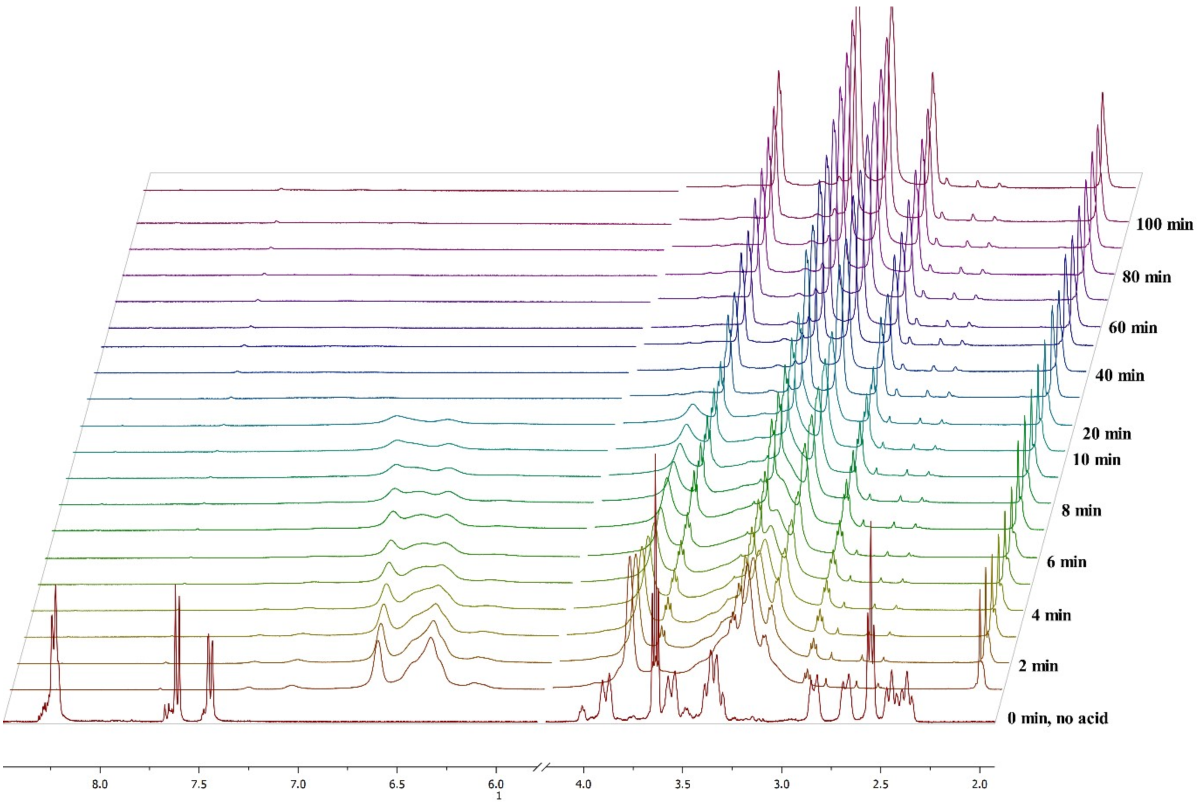

Figure S14. NMR measurement of $\mathbf{K_21a}$ in deuterated acetic acid buffer at pH 4.

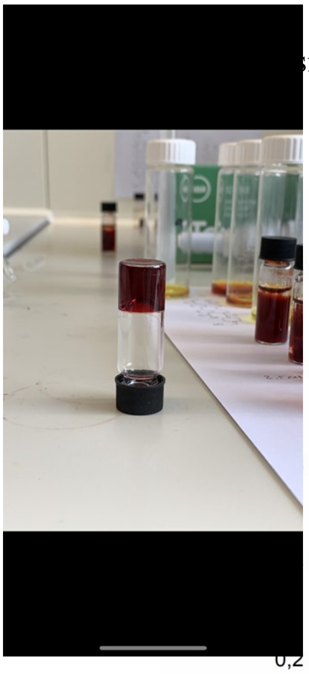

[illegible]sion experiment of 2.5 mM $\mathbf{K_21a}$ in 0.25 M AcOH buffer of pH 4, 105 minutes after acidification.

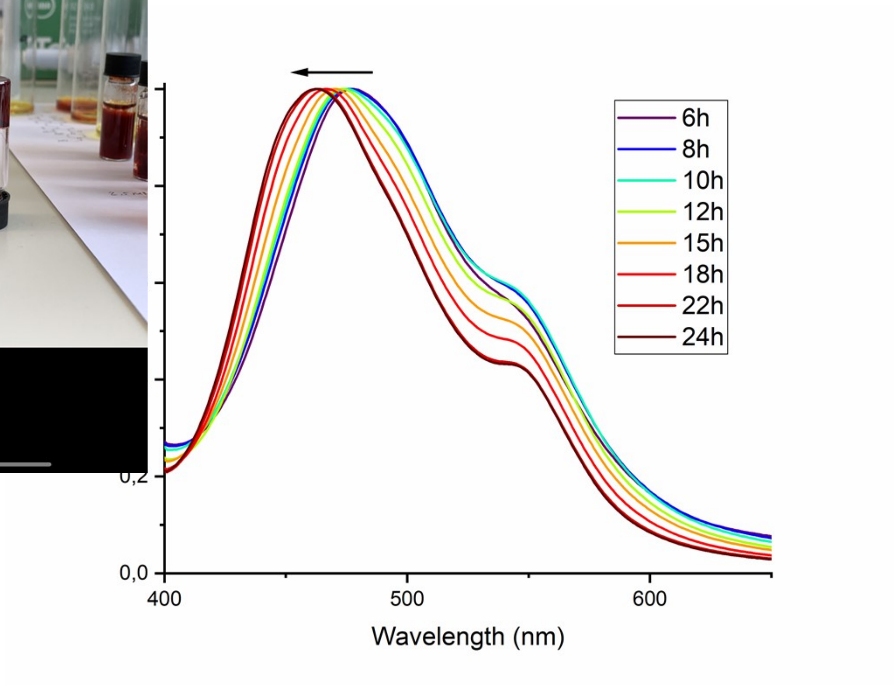

Figure S16. Normalised absorption of the gel formation of $\mathbf{K_21a}$ (0.23 mM) with 0.05 M freshly prepared GdL solution.

UV-Vis and NMR spectra of $K_2$1a and $K_2$1b

The absorption spectra of **$K_2$1a** and **$K_2$1b** (Figure S17) were identical, show clear vibronic structure and are very similar to reported spectra of a similar amic acid salt.[3]

The ^{1}H and ^{13}C NMR spectra of compound **$K_2$1a** in CD_3OD are shown in Figures S18-S21, those of **$K_2$1b** in Figures S22- S25. In the proton NMR of **$K_2$1a** the presence of two isomers is not apparent from the aromatic part of the spectrum, most likely due to strong overlap. In the aliphatic region the methylene units of the hydroxyethyl groups attached to the piperazine ring are visible as two sharp triplets at 2.59 and 3.67 ppm. The protons on the piperazine rings exhibit a complex coupling pattern. Integration of the multiplet resonances demonstrates that all 8 protons attached to the piperazine ring have different chemical shifts and are individually visible. This observation indicates that both the inversion of the six-membered piperazine ring and the rotation over the CO-NR_1R_2 amide bond[2] at the peri-position are slow on the NMR timescale. The carbon NMR spectrum reveals that **$K_2$1a** indeed is a mixture of cis and trans isomers in a ~1:1 ratio. For the carboxylate and amide carbonyl carbons, at 175.7 and 171.3 ppm, respectively, double resonances are observed. Furthermore, the total number of resonances in the aromatic region is 20, as expected for a mixture of cis and trans isomers, see Figures S19 and S24.

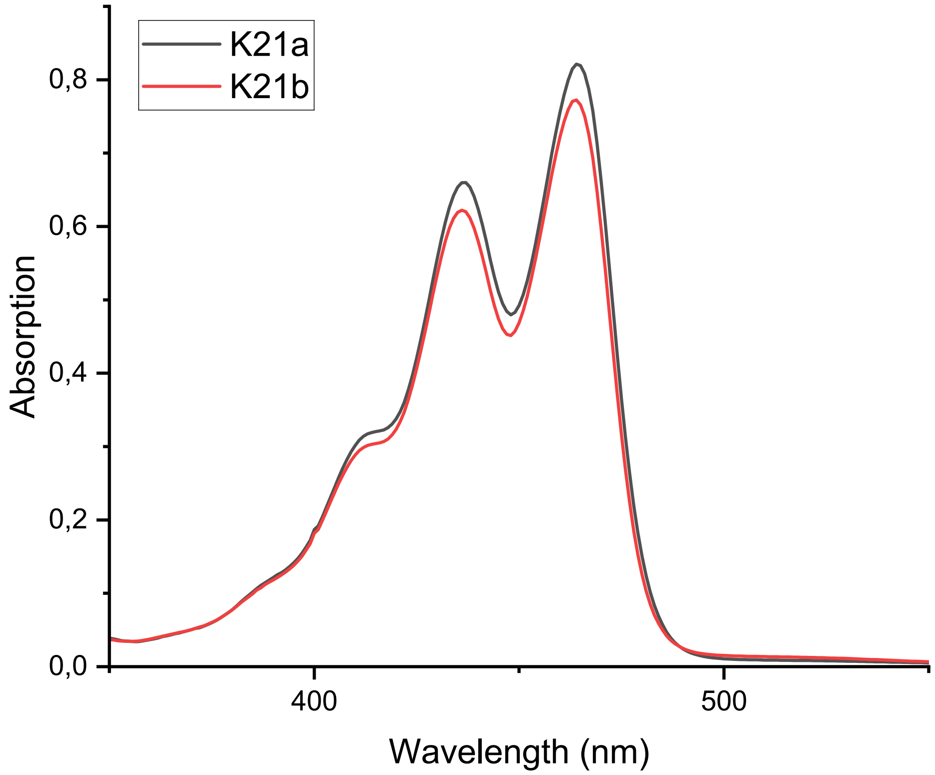

Figure S17. Absorption spectra of **$K_2$1a** (black) and **$K_2$1b** (red) in water. Gelator concentrations are ~ 0.025 mM.

NMR spectra

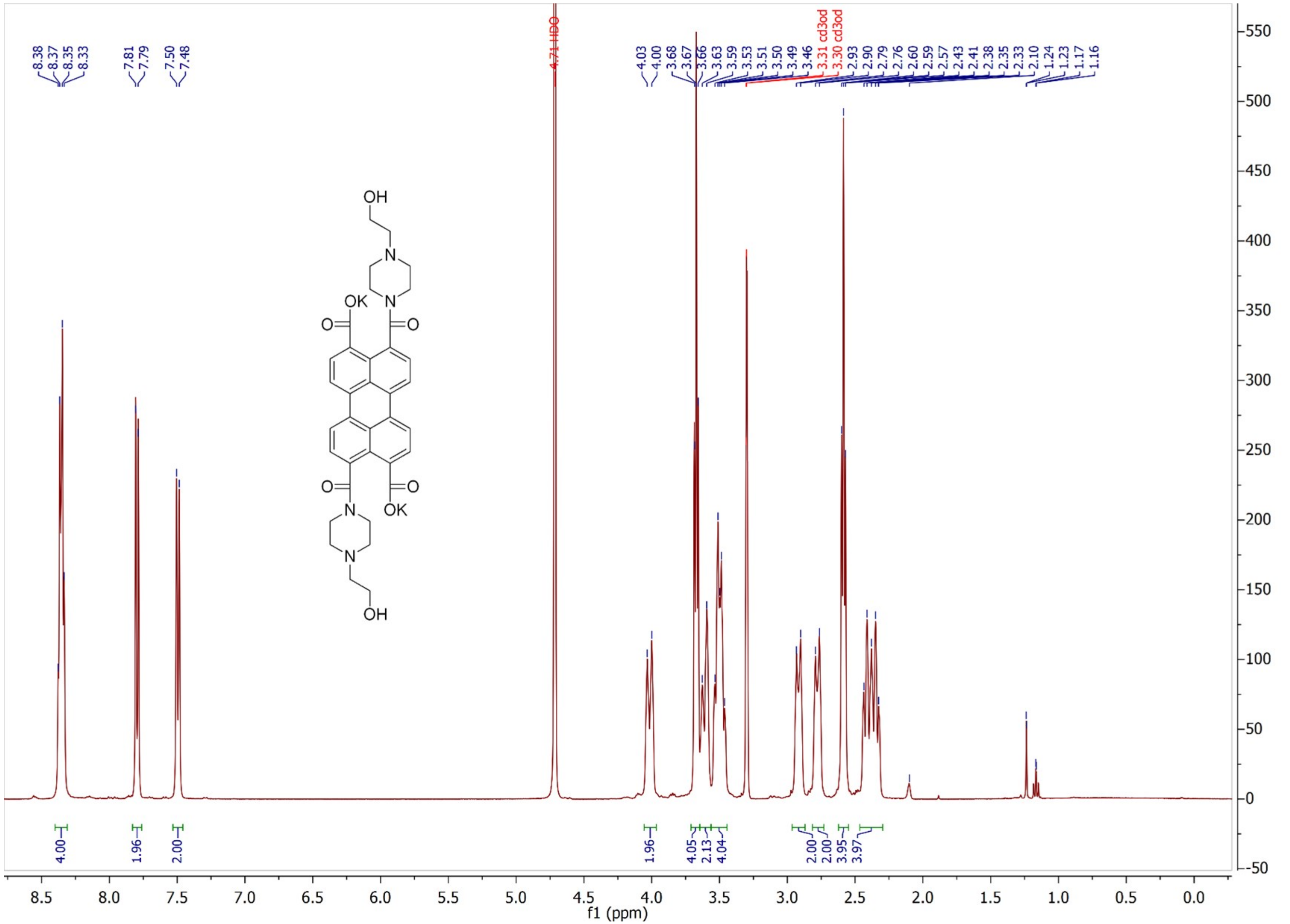

Figure S18. ^{1}H-NMR spectrum of $\mathbf{K_2 1a}$ in CD_3OD.

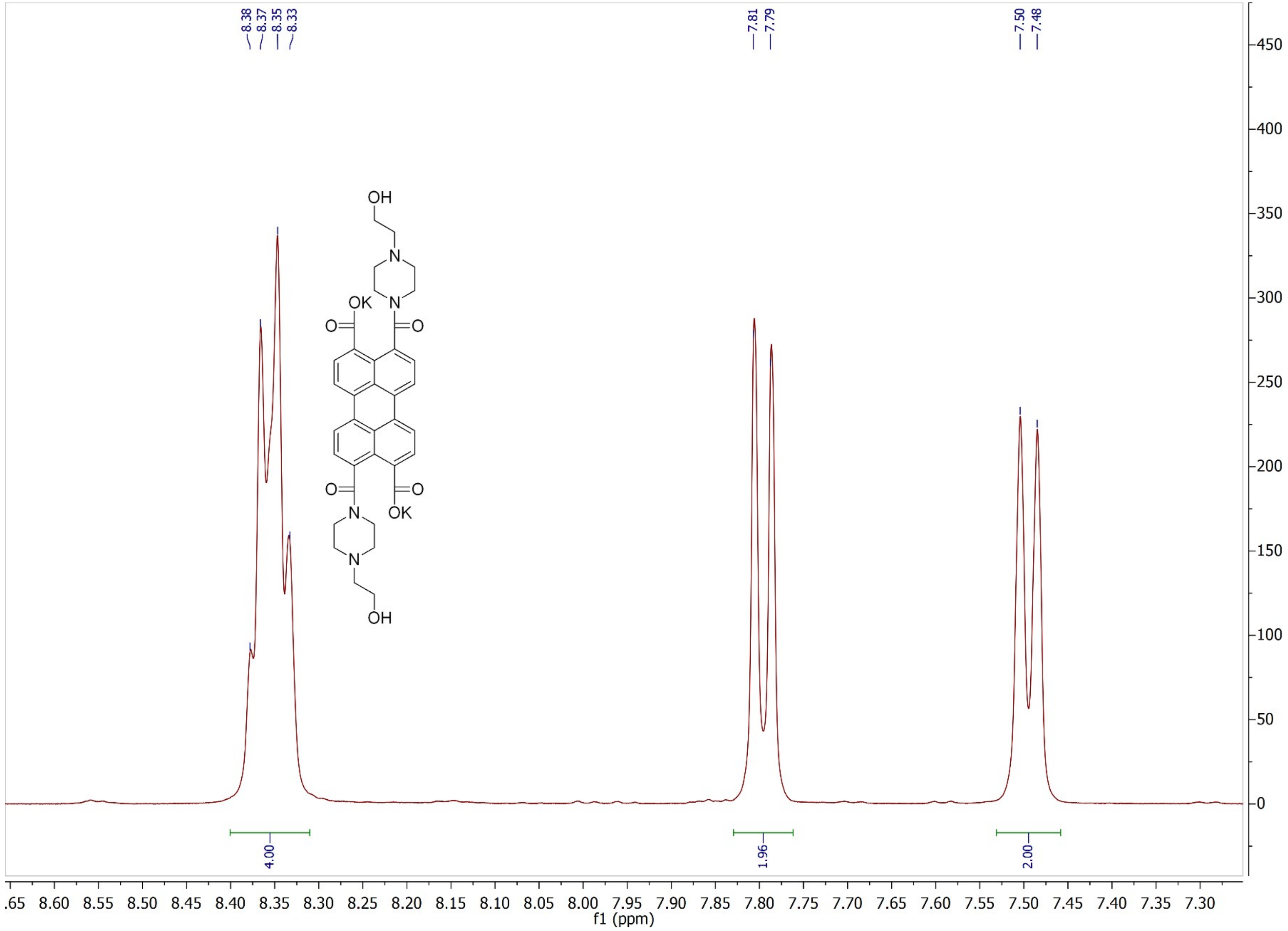

Figure S19. ^{1}H-NMR spectrum of **$K_2$1a** in CD_3OD, aromatic region.

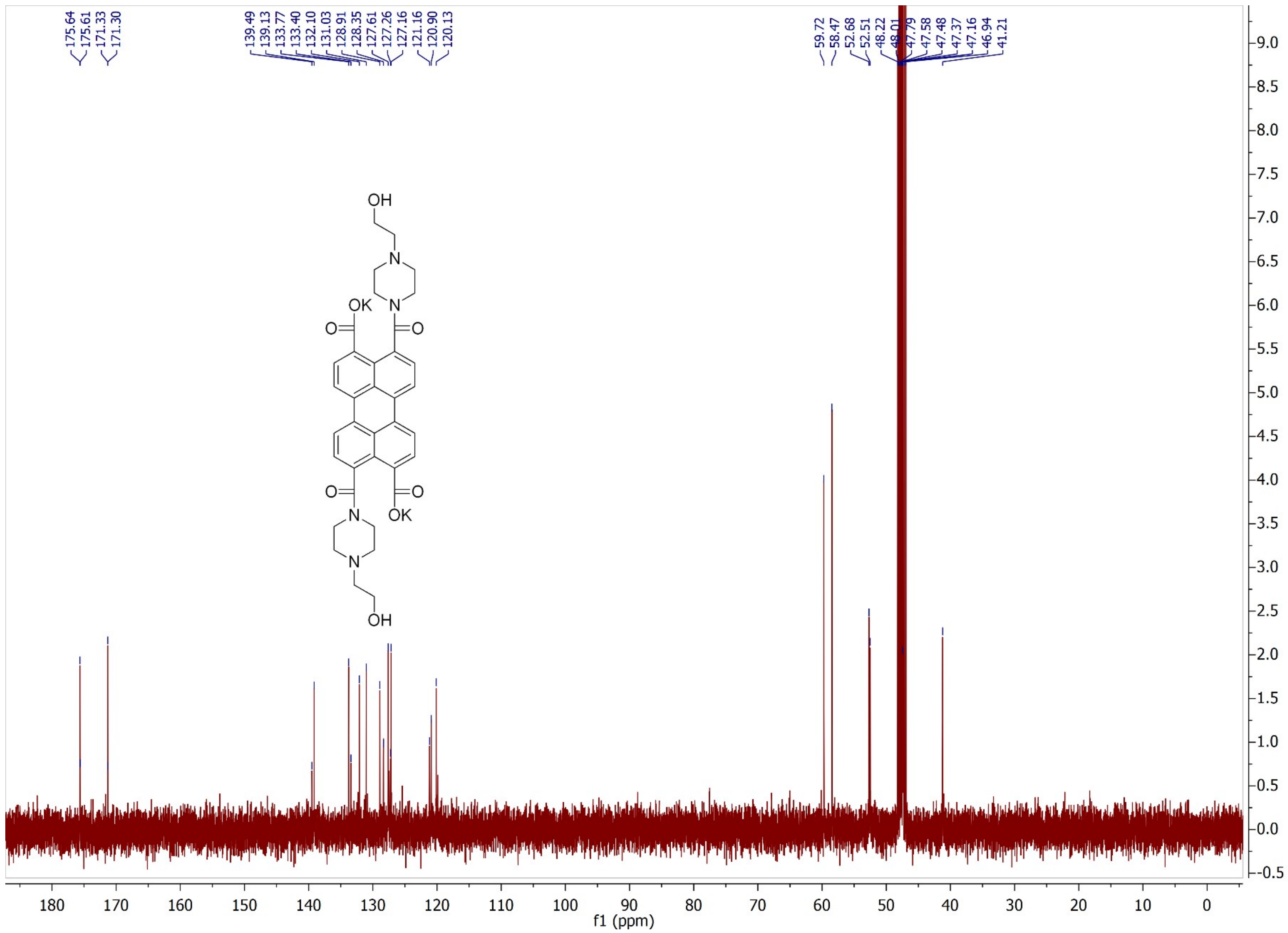

Figure S20. ^{13}C-NMR spectrum of **K$_2$1a** in CD$_3$OD.

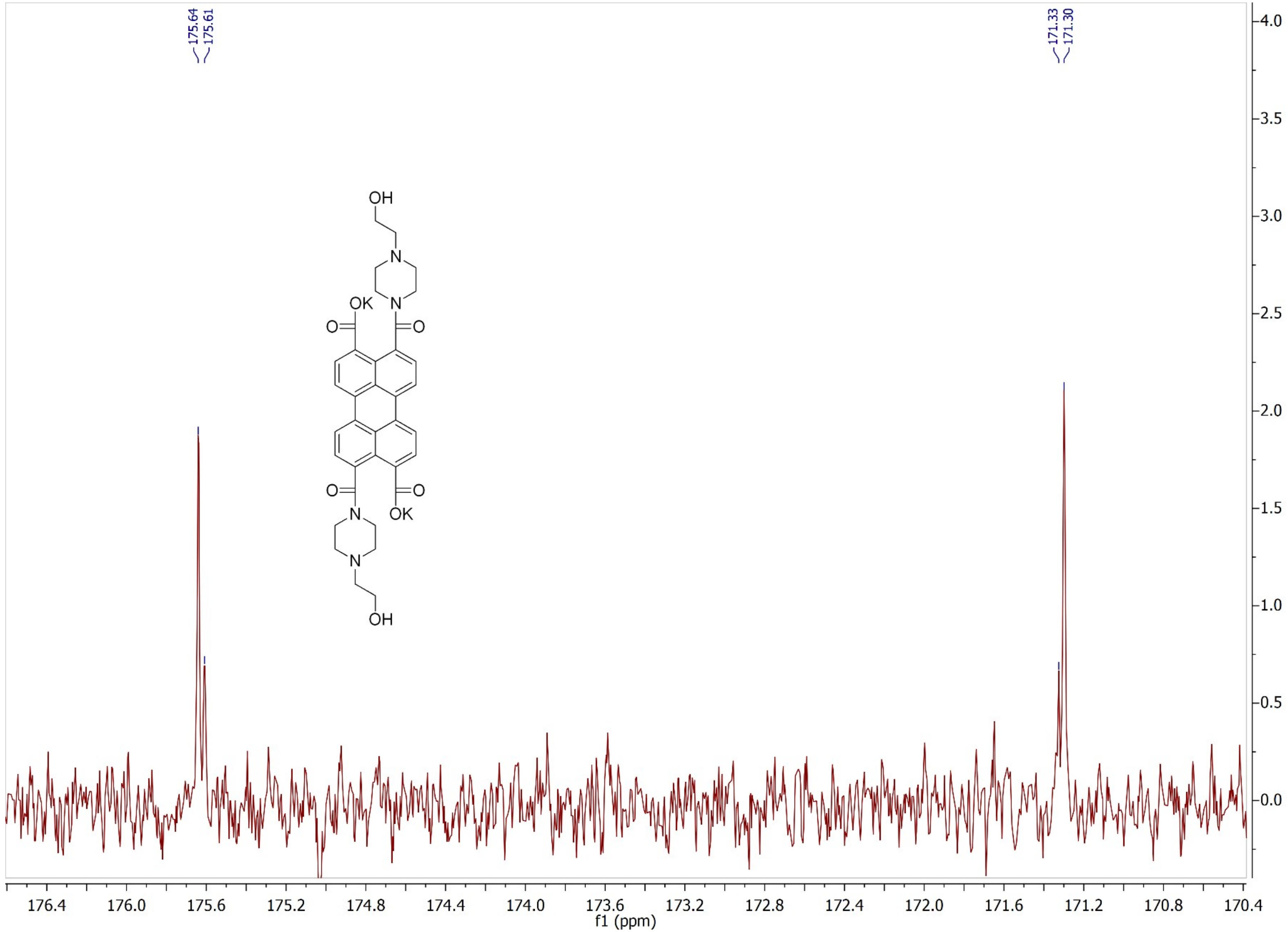

Figure S21. ^{13}C-NMR spectrum of $\mathbf{K_2 1a}$ in CD_3OD, carbonyl region.

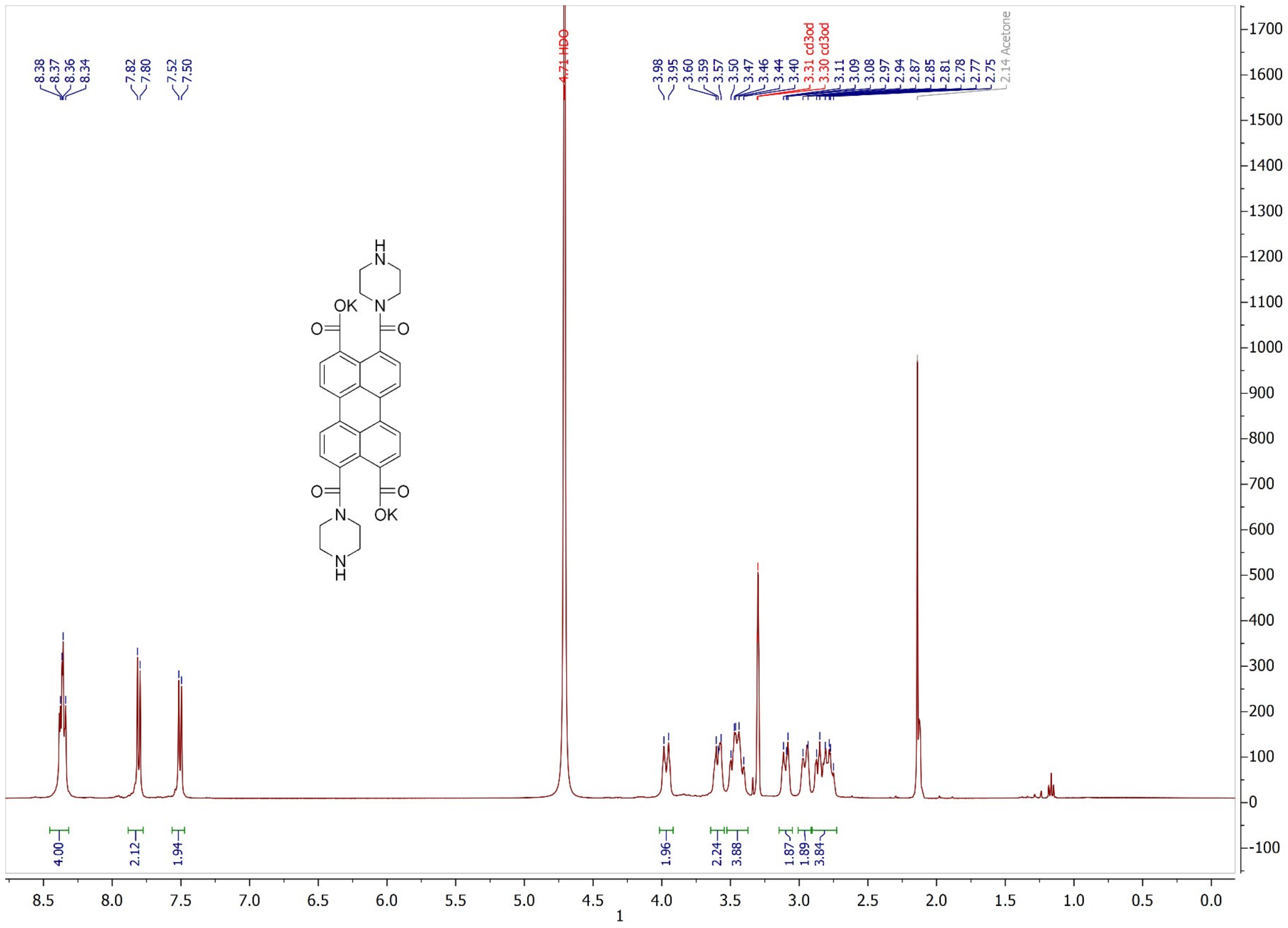

Figure S22. ^{1}H-NMR spectrum of $\mathbf{K_2 1b}$ in CD_3OD.

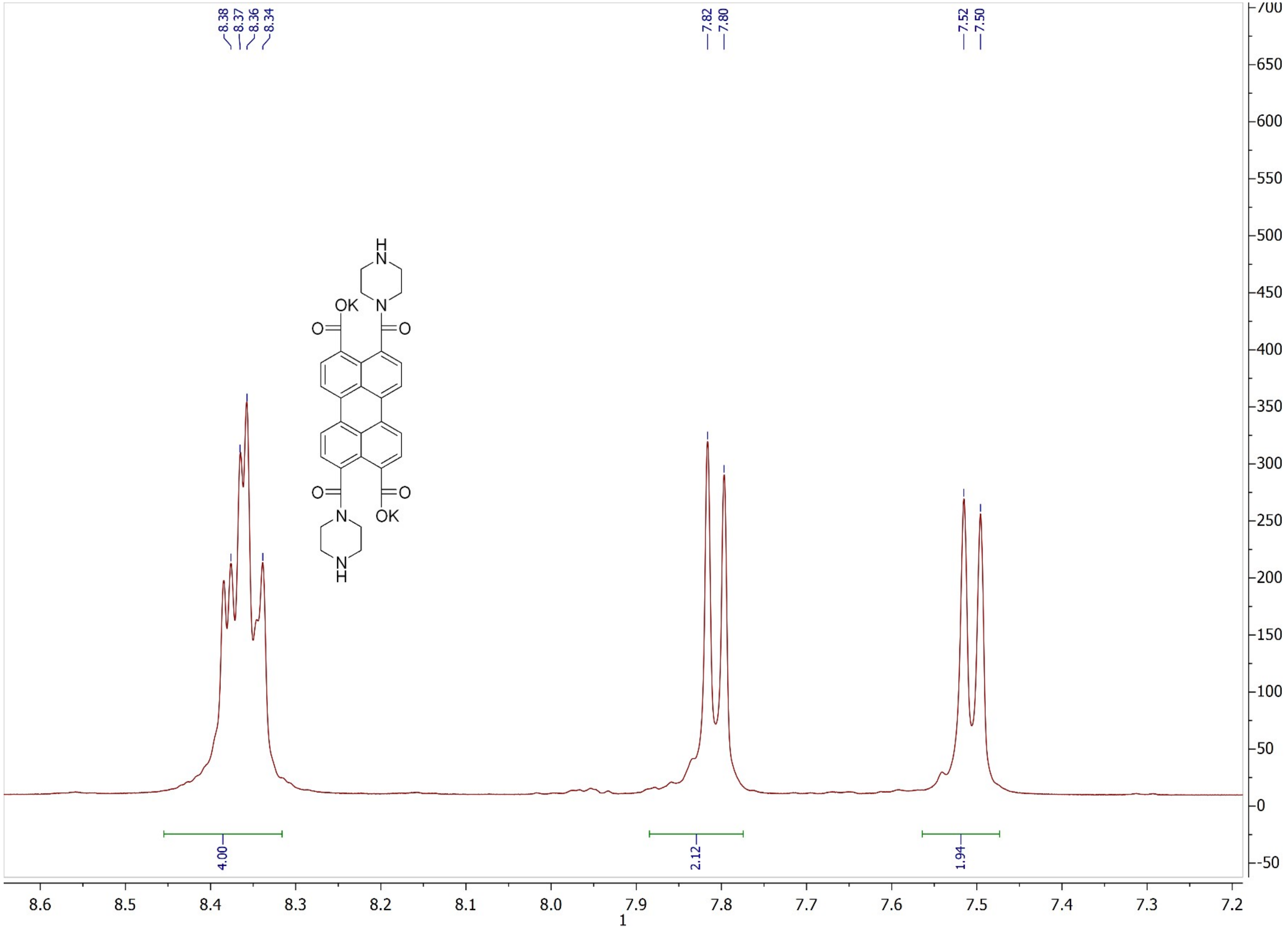

Figure S23. ^{1}H-NMR spectrum of $\mathbf{K_2 1b}$ in CD_3OD, aromatic region.

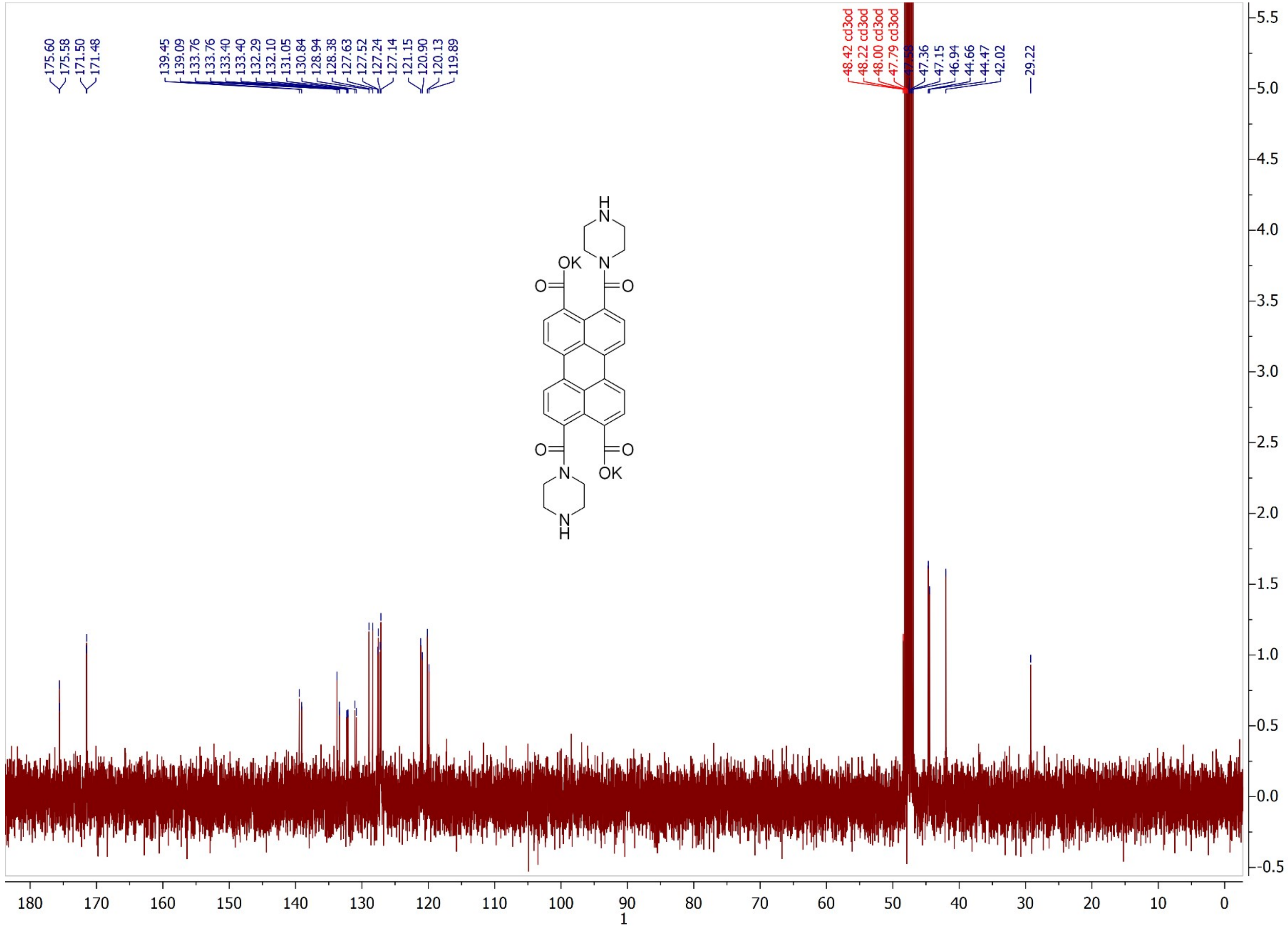

Figure S24. ^{13}C-NMR spectrum of $\mathbf{K_21b}$ in CD_3OD.

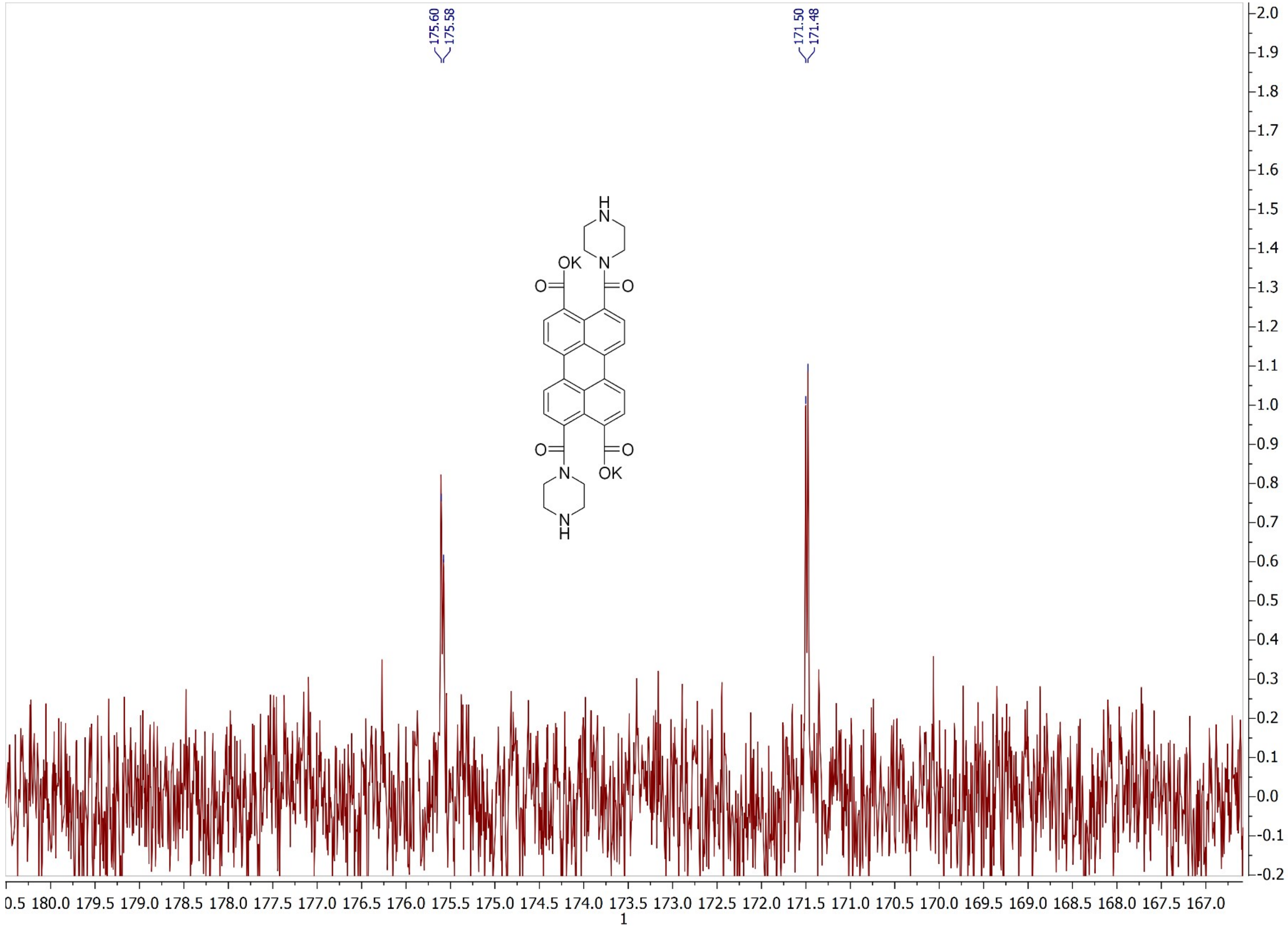

Figure S25. ^{13}C-NMR spectrum of $\mathbf{K_2 1b}$ in CD_3OD, carbonyl region.

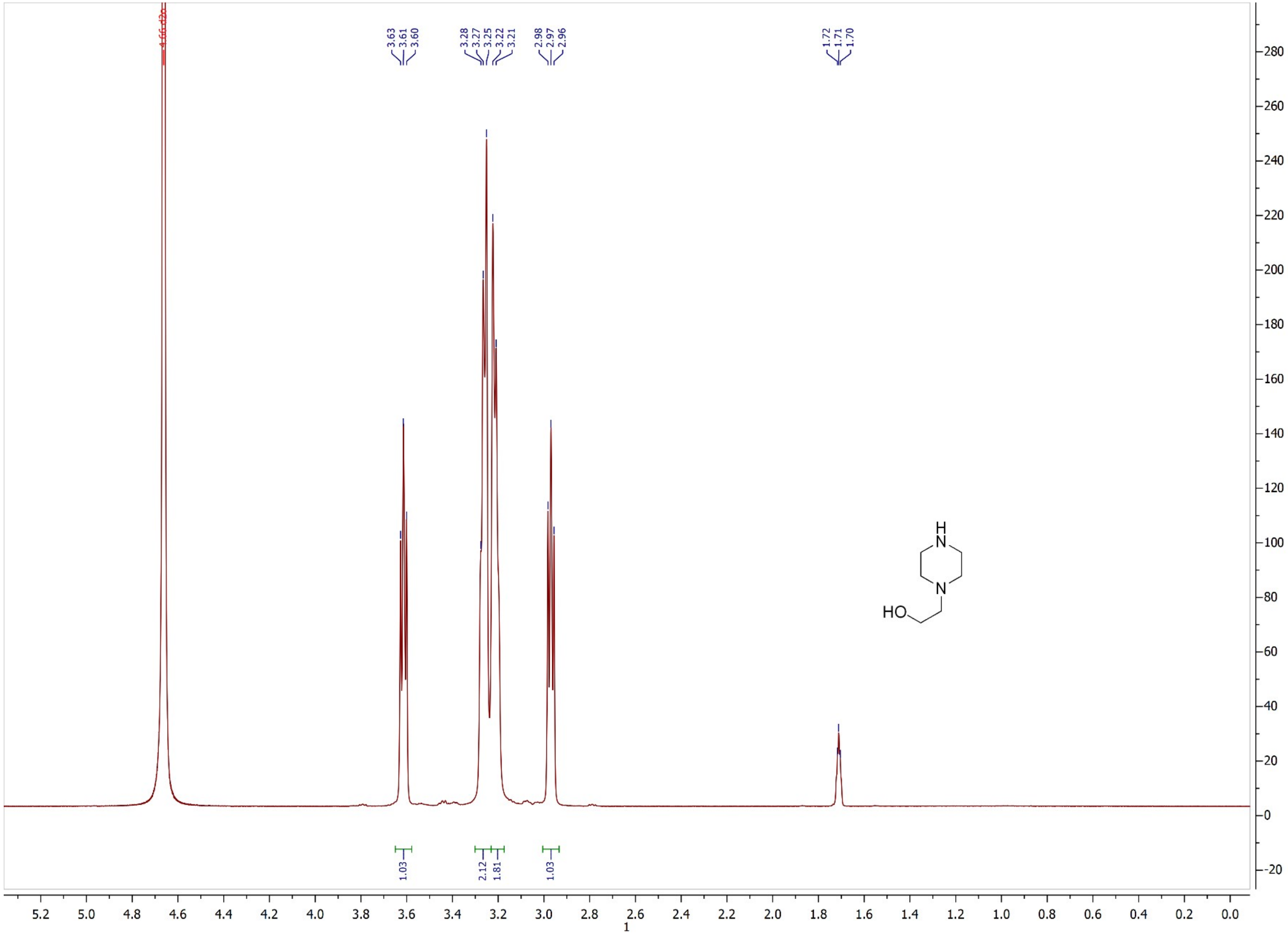

Figure S26. ^{1}H-NMR spectrum of 1-(2-hydroxyethyl)piperazine (**4a**) in D_2O and AcOD at pH 4.

References

(1) Zhang, Q.; Zhang, P.; Li, S.; Fu, C.; Ding, C. Aggregation-disaggregation-switched sensing strategy for copper(II) ion and histidine in aqueous solution and living cell imaging *Dyes and Pigments*, **2019**, *171*, 107697

(2) Langhals, H.; Karolin, J.; Johansson, L. B.-Å. Spectroscopic Properties of New and Convenient Standards for Measuring Fluorescence Quantum Yields. *J. Chem. Soc. Faraday Trans.* **1998**, *94* (19), 2919–2922. https://doi.org/10.1039/a804973d.

(3) Kwakernaak, M. C.; Koel, M.; van den Berg, P. J. H.; Kelder, W. M.; Jager, W. F. Room temperature synthesis of perylene diimides facilitated by high amic acid solubility Org Chem Front 2022, **9**, 1090-1108
